# Application of spatially robust stereo‐BRUV sampling for quantifying fish assemblages in UK marine protected areas

**DOI:** 10.1002/eap.70104

**Published:** 2025-09-12

**Authors:** Owen M. Exeter, Annette C. Broderick, Xavier A. Harrison, Francesco Garzon, Sarah Morcom, Ricky Pender, Trudy Russell, Ian Saunders, Paul J. Somerfield, Kate Sugar, Colin Trundle, Julie Webber, Tom Hooper, Kristian Metcalfe

**Affiliations:** ^1^ Centre for Ecology and Conservation, Faculty of Environment, Science and Economy University of Exeter Penryn Cornwall UK; ^2^ Hatherly Laboratories, University of Exeter Exeter UK; ^3^ Isles of Scilly Inshore Fisheries and Conservation Authority St. Mary's Isles of Scilly UK; ^4^ Natural England York UK; ^5^ Plymouth Marine Laboratory Plymouth Devon UK; ^6^ Fishtek Marine Dartington Devon UK; ^7^ College of Marine Science and Aquatic Biology, University of Khorfakkan Sharjah UAE; ^8^ Sharjah Marine Sciences Research Centre (SMSRC), University of Khorfakkan Sharjah UAE

**Keywords:** baited remote underwater video systems, BRUVS, island biogeography, marine ecosystems, marine monitoring, marine protected areas

## Abstract

Marine protected areas (MPAs) often lack adequate data on the status of marine assemblages to support evidence‐based management. Stereo baited remote underwater video (BRUV) systems offer a powerful, low‐cost tool for collecting ecological data, yet they remain underutilized in the North East Atlantic, especially compared to more invasive methods such as fisheries surveys. Here, we demonstrate how a spatially comprehensive stereo‐BRUV survey can generate benchmark data to support MPA management at an ecosystem scale, using an ecologically distinct oceanic archipelago as a case study. The archipelago's habitats were found to support high abundances of regionally targeted commercial species, including benthic catsharks (Scyliorhinidae) and European spiny lobster (*Palinurus elephas*), with ~12,000 individuals recorded representing 64 species and 44 families. Deeper, topographically complex reefs were found to support higher levels of richness and biomass, with sediment‐specific increases in depth also driving demersal abundance. Stereo technology was additionally able to provide body size data for 43 species, with remoteness and shelter from exposure found to be common drivers of increased body size for indicator taxa. Survey results represent a contemporary benchmark for measuring changes in local MPA management, fisheries practices, and climate change impacts. The results also illustrate how spatially robust sampling methods and stereo‐BRUV systems can facilitate more holistic, fisheries‐independent data collection in UK and European waters.

## INTRODUCTION

Marine ecosystems are increasingly being impacted by human activities (Halpern et al., 2019; O'Hara et al., 2021), with destructive fishing practices, pollution, and climate change (Anthony et al., 2008; Wernberg et al., 2011), leading to habitat degradation, species decline (Christiansen et al., 2020; Dulvy & Reynolds, 2002; Pacoureau et al., 2021), and food insecurity for coastal communities (Belhabib et al., 2015; Sumaila & Vasconcellos, 2000). To mitigate against these impacts, a variety of spatial management initiatives have been explored, including marine spatial planning (MSP) and ecosystem‐based management (EBM) (Douvere, 2008), aiming to improve the sustainability of human activities and alleviate pressures on marine ecosystems.

Marine protected areas (MPAs) have become one of the primary tools for implementing spatial conservation management at sea (Grorud‐Colvert et al., 2021). International treaties and initiatives such as the Convention on Biological Diversity Kunming–Montreal Global Biodiversity Framework have targeted 30% of the world's oceans to be conserved by 2030, driving an increase in designations globally (Sala et al., 2018; Thomas et al., 2014). When properly designed and effectively enforced (Canty et al., 2024), MPAs have been shown to increase fish biomass (Aburto‐Oropeza et al., 2011; Goetze et al., 2021; Sala & Giakoumi, 2018), conserve threatened species (Davidson & Dulvy, 2017; Roberts et al., 2021), allow recovery of marine habitats (Davies, Holmes, Attrill, & Sheehan, 2022; Davies, Holmes, Bicknell, et al., 2022; Medrano et al., 2020; Sheehan et al., 2013), and potentially buffer the impacts of climate change (Ling & Johnson, 2012). They have also been recognized for having secondary benefits to coastal communities and fisheries through processes such as the spillover of adult fish (Lenihan et al., 2021; Medoff et al., 2022), larval dispersion (Christie et al., 2010; Green et al., 2015), and alternative income opportunities (Nowakowski et al., 2023; Pham, 2020).

The effectiveness of MPAs and other spatial management interventions is, however, often limited by poor design (Agardy et al., 2011; Conners et al., 2022), inadequate management (Claudet et al., 2020; Sala et al., 2018), and insufficient long‐term monitoring (Addison, 2011). Many of these issues stem from a lack of basic data on the diversity, distribution, and abundance of habitats and species that MPAs are designated to protect (Espinoza et al., 2020; Metcalfe et al., 2013; Rees et al., 2020). This paucity of data is often attributed to the difficulties and costs associated with collecting marine data, particularly in remote locations, for mobile taxa, and in temperate systems due to weather and visibility (Noble‐James et al., 2023; Wilding et al., 2017). Without adequate baseline or benchmark data, however, the ability to detect changes in the health and status of marine communities, and therefore track MPA effectiveness in the face of changing management policies, human pressures, or climate change, remains challenging (Ban et al., 2023; Espinoza et al., 2024). Developing cost‐effective approaches for assessing the marine environment, particularly identifying areas of elevated diversity and essential habitat, is therefore essential for evidence‐based marine management (Espinoza et al., 2020; Hyvärinen et al., 2021).

Recent advances in remote camera technologies have improved the accessibility and cost of easily repeatable survey methods for marine monitoring (Bicknell et al., 2016; Perkins et al., 2021). Baited remote underwater video (BRUV) systems have proven particularly effective for assessing the health and status of marine assemblages (Espinoza et al., 2020; White et al., 2013), species–habitat associations (Brown et al., 2022), and management interventions across various marine systems globally (Albano et al., 2021; Goetze et al., 2021). BRUV systems are also considered low impact (Whitmarsh et al., 2017), making them more compatible with MPA objectives than invasive methods such as scientific fishing surveys (Trenkel et al., 2019). The advancement of stereo BRUV (dual camera) technology also allows for the recording of length data that can be used to derive biomass estimates, thereby ensuring that outputs can be aligned with fisheries‐dependent data (Letessier et al., 2024; Meeuwig, 2021). Additionally, BRUV systems are considered safe and applicable across a range of habitats (Whitmarsh et al., 2017), including those less suitable for traditional underwater visual census dive surveys (Baremore et al., 2023; Bouchet & Meeuwig, 2015), which can be resource intensive in terms of personnel, cost, and time and limited by depth and safety considerations (but are known to record smaller and more cryptic species compared to BRUV systems; Lowry et al., 2012). Despite their low cost, accessibility, and repeatable nature, BRUV application has been limited to a relatively small number of sites and surveys in UK waters to date (i.e., Clark et al., 2024; Davies, Holmes, Attrill, & Sheehan, 2022; Davies, Holmes, Bicknell, et al., 2022; Unsworth et al., 2014). Wider incorporation of BRUV systems into UK marine monitoring programs has the potential to generate comprehensive modern benchmarks which are often lacking for existing MPAs (Hill et al., 2018). They also have the potential to improve knowledge of local environmental and anthropogenic drivers of marine assemblages (Brown et al., 2022; Furness & Unsworth, 2020) and allow regional or national assessments of MPA effectiveness if methods are standardized (Canty et al., 2024; Goetze et al., 2021).

Here, we present the largest scale stereo BRUV survey conducted to date in the UK across the Isles of Scilly archipelago and its MPAs. Despite being considered a regionally unique biodiversity hotspot (Lewis et al., 2008; Warwick et al., 2003), there exist limited data on the local diversity and abundance of marine teleost fish, elasmobranchs, and macroinvertebrates (Exeter et al., 2024). Understanding the current health of the archipelago at an ecosystem level therefore remains challenging, and the impacts of novel threats such as habitat regime shifts from invasions (Barrett et al., 2007) and climate change (Smale et al., 2019), noise pollution, and shifts in fishing practices are hard to quantify. Long‐term application of BRUV surveys can therefore address this knowledge gap and complement ongoing annual monitoring of other conservation features (i.e., seabird colonies and seagrass bed health; Exeter et al., 2024). This study therefore adopts a spatially balanced probabilistic sampling approach to survey the archipelago's mobile marine assemblages and: (1) establish a modern benchmark of local marine species from which future changes can be measured against; (2) explore the spatial and environmental drivers of species richness, abundance, diversity, biomass, and indicator species body length; and (3) evaluate the effectiveness of large‐scale stereo BRUV for surveying in UK waters. Findings from this study will contribute to the archipelago's future MPA management and enhance the knowledge base of drivers of marine diversity within isolated archipelago systems. Given BRUV systems are an underutilized monitoring tool in the domestic English waters, this study also highlights the value of spatially comprehensive stereo BRUV surveys for UK MPA monitoring.

## METHODS

### Study area

**FIGURE 1:**
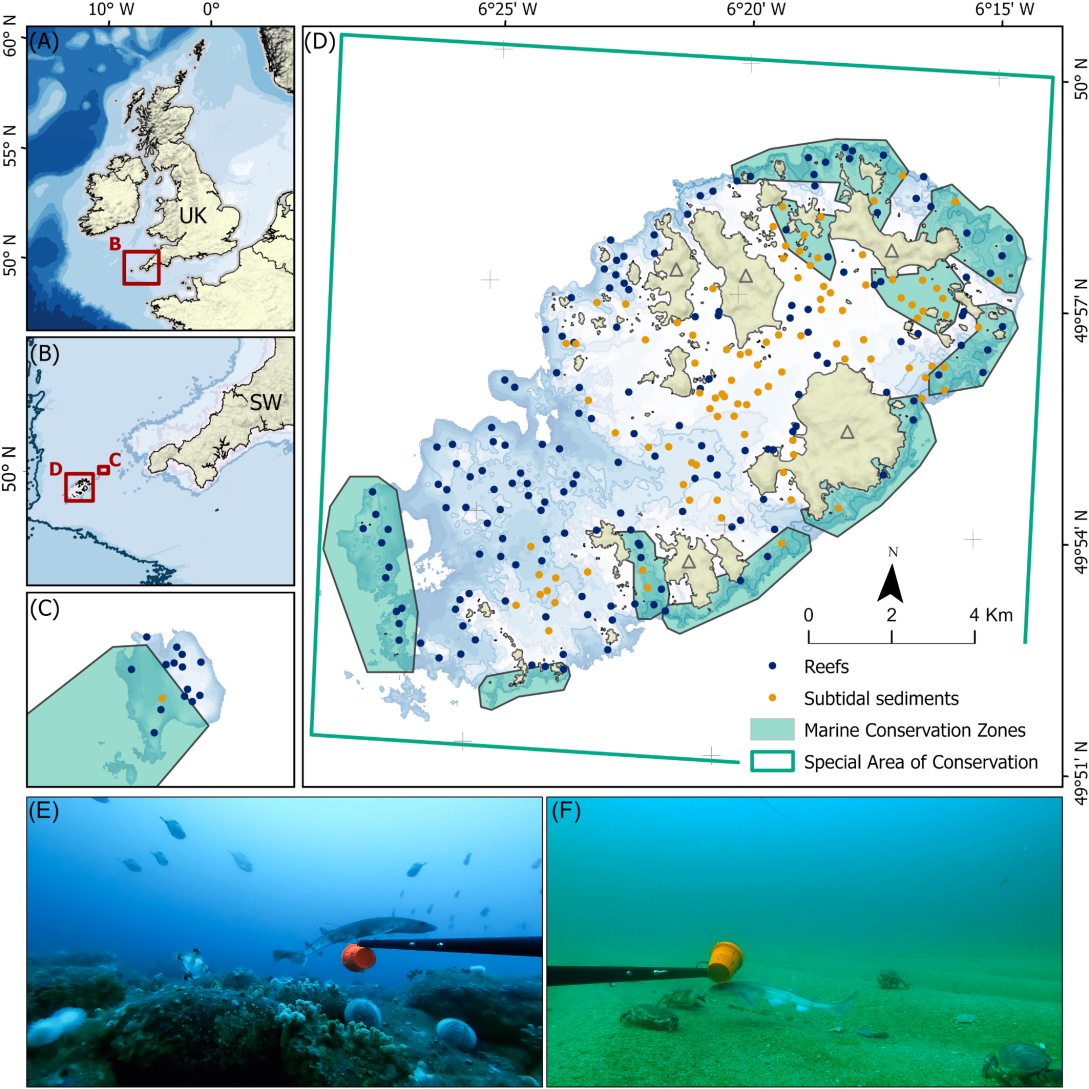
Map of study area with (A) the United Kingdom and surrounding bathymetry (Global Bathymetric Chart of the Oceans (GEBCO) data) with an inset (red box) indicating the extent of (B) the Southwest UK peninsula and insets of (C) the Seven Stones Reef, and (D) the Isles of Scilly inshore zone, with bathymetry <40 m highlighted and inhabited islands indicated by a gray arrow at their centroid. GRTS survey locations indicated by blue (reef) and orange (sediment) points. Marine Conservation Zone boundaries (green solid polygons) and the Special Area of Conservation (green outline) are also shown, with examples of designated feature habitat types Reefs (E) and Subtidal sediments (F). Photo credits for (E) and (F): Owen M. Exeter.

The Isles of Scilly (49.9310° N, 6.3258° W) is an oceanic archipelago situated over 20 nm off the Southwest United Kingdom coast (Figure 1). The archipelago supports a regionally unique mosaic of warm and cold temperate coastal and pelagic habitats and species attributed to its position on the European continental shelf coupled with high exposure to oceanic currents (Lewis et al., 2008; Warwick et al., 2003). This diversity includes communities of nationally rare sponges, cold‐water corals, and macroalgae (Lewis et al., 2008; Pikesley et al., 2016), some of the largest and healthiest seagrass beds in UK waters (Bull & Kenyon, 2021; Jones & Unsworth, 2015), extensive pelagic plankton communities (Cunningham et al., 2003), a breeding bird assemblage (>20,000 breeding seabirds at designation) (Heaney et al., 2008), and a wide variety of crustacean, teleost fish, and elasmobranch species (Delaval et al., 2023; Herdson, 2010; Whomersley et al., 2018).

This biodiversity has made the Isles of Scilly an important research and monitoring site (Fowler & Pilley, 1992; Warwick et al., 2003) and resulted in a variety of spatial management measures (MPAs) being designated (Figure 1). These include a Special Area of Conservation (SAC), a Special Protection Area, and a network of smaller Marine Conservation Zones that combined protect a diverse range of habitats and species (Exeter et al., 2024). As a result of the archipelago's biogeography, management, and small resident human population, local ecosystems are perceived as being in a comparatively pristine condition, relative to the highly degraded wider Northeast Atlantic marine region (Halpern et al., 2008). As such, the archipelago can act as an important reference site of what a contemporary, near‐natural seascape may be for the region. However, there currently exists limited knowledge on many of the marine species present. Data are notably lacking on the diversity and distribution of mobile marine fish, elasmobranchs, and crustacean species, particularly in exposed locations and deep‐water reefs that are difficult to access with traditional survey methods such as dive surveys (Axelsson et al., 2014; Exeter et al., 2024).

### Survey design and data collection

BRUV survey locations were generated using a generalized random tessellation stratified (GRTS) design (Stevens & Olsen, 2004), using the “spsurvey” package (Dumelle et al., 2023) in R (v 4.1.1) and R Studio (R Core Team, 2021). GRTS sampling generates a statistically robust, but spatially balanced set of random locations (Smith et al., 2017) while allowing for stratification between zones of interest (Bouchet et al., 2020; Currey‐Randall et al., 2021; Hill et al., 2014). Survey locations were generated for 300 deployments spanning a depth range of 0–40 m (Figure 1) account for the depth rating of the camera housing (60 m) and buffer for tidal range, units shifting post deployment, and accuracy of bathymetry data. A minimum 200‐m distance between deployments was specified to avoid potential overlap of bait plumes and the probability of recapturing individuals in neighboring samples if deployed on the same sampling day (Whitmarsh et al., 2017). The survey design was stratified by the spatial extent of marine (subtidal) habitat features within the Special Area of Conservation MPA (data provided under license from Natural England; Marine Evidence Base [Internal] dataset 2021). This resulted in 200 deployment locations for “Reefs,” comprising 100 deployment locations within both “Circalittoral rock” and “Infralittoral rock” sub‐feature habitat types (Figure 1). A further 100 deployments were generated for “Sandbanks which are slightly covered by sea water all the time” (henceforth “Subtidal sediments”) habitats, comprising 50 deployment locations within the “Subtidal seagrass beds” sub‐feature habitat type and 50 deployments combining the sub‐features: “Subtidal coarse sediment,” “Subtidal mixed sediments,” and “Subtidal sand” (henceforth “Sediments”) (Figure 1). Each deployment was surveyed once, across months surrounding the boreal summer (late May–September) of 2022 and 2023. Effort was spread evenly between years (*n* = 150 per annum), and across habitat types throughout the survey season.

Stereo carbon‐fiber BRUV manufactured by Blue Abacus (Letessier et al., 2024) was the primary system used in this study, with a mono (single camera) PVC system also used for a small number of deployments, notably in areas where deployments were challenging (i.e., strong currents). Units comprised a single (*n* = 2) or pair (*n* = 5) of action cameras (GoPro Hero 4, 8, and 9 s, all 30 fps 1920 × 1080‐pixel resolution, medium field of view) and were baited with 250 g of locally caught crushed Atlantic mackerel (*Scomber scombrus*), shown to be effective for BRUV sampling in UK waters (Bicknell et al., 2019; Blampied et al., 2022; Clark et al., 2024; Jones et al., 2020). Soak duration lasted 70 min from when the BRUV system landed on the seabed to ensure that a standardized 60 min of footage would be available for analysis (Unsworth et al., 2014; Whitmarsh et al., 2017). Deployments were conducted in a two‐hour window either side of slack water to reduce the influence of variation in tidal flow on the data (Taylor et al., 2013).

### Data processing

Deployments were analyzed by a single observer for 60 min from the time the unit settled on the seabed, using the SeaGIS EventMeasure software v. 6.06 (www.seagis.com.au). To standardize between mono and stereo systems, only one camera was used for species identification during video processing. Observed individuals were recorded to the lowest taxonomic level possible. For each deployment, the true habitat type was visually determined once the BRUV system had settled on the seabed and assigned to either “Reefs” or “Sediments” and their associated sub‐habitat type “Circalittoral rock,” “Infralittoral rock,” “Subtidal seagrass beds,” and “Subtidal sediments.”

### Species richness, abundance, diversity, and biomass

For each deployment, the following ecological indices were calculated: species richness, relative abundance, Shannon–Weiner Diversity Index, and total biomass. Species richness corresponds to the total number of unique species observed per 60‐min deployment. Relative abundance corresponds to the maximum number of individuals per species recorded within any one frame per deployment (MaxN) and is the standardized abundance metric used in BRUV studies to prevent double counting of individuals (Langlois et al., 2020). Species richness and relative abundance values were subsequently subset for further analyses to only include demersal‐ and benthic‐associated teleost fish, elasmobranchs, and macroinvertebrates (henceforth “demersal”). This was due to the hyperabundant, but sporadic nature of shoaling pelagic‐neritic species, and known limitations of benthic BRUV systems for accurately recording pelagic fish (Clarke et al., 2019). While species such as *Pollachius pollachius* and Ammodytidae spp. can also occur in hyperabundant shoals, they are classified as reef or seabed associated species and form important parts of demersal food webs, so were retained for analysis. Shannon–Weiner Species Diversity Index (henceforth “diversity”) was calculated for all species using the R package vegan (Oksanen et al., 2024) and provides a standardized metric incorporating both species richness and abundance.

For biomass (in kilograms) values, species lengths were first calculated for all teleost fish, elasmobranch, and crustacean species using the Event Measure software. Lengths were measured for all stereo deployments within frames corresponding to MaxN to avoid double measuring individuals (Hill et al., 2018). Only individuals that could be accurately measured (i.e., close to camera and presenting a side profile) were retained. If no individuals could be measured around the time of MaxN (i.e., due to obstructions or not presenting a side on profile to measure), then lengths were measured within frames corresponding to the next highest MaxN. Caudal lengths (CL) or total lengths (TL) (depending on fin morphology) were measured for all teleost fish and elasmobranch species, carapace length for both *Homarus gammarus* and *Palinurus elephas*, and carapace width for *Cancer pagurus* and *Maja brachydactyla*, to compare against local commercial and recreational minimum landing size (MLS) byelaws. Species length–weight relationships were calculated using geometric mean values of studies listed on the FishBase and SeaLifeBase repositories (Meeuwig, 2021). For species with large distributions (i.e., *Galeorhinus galeus*), only studies from the Northeast Atlantic and Mediterranean Sea were used to inform the biomass calculations. For each sample, the mean length (in millimeters) was used to generate a mean weight (in grams) per species and multiplied by the species‐specific MaxN value for each deployment. If a length for a species occurring in a deployment could not be accurately measured (i.e., due to an obstruction) or if the BRUV system was a mono unit, a proxy length was applied (Barley et al., 2017) to allow a comparable total biomass (in kilograms) estimate for each deployment. Proxy lengths were selected using the following steps adapted from Meeuwig et al. (2021) in order: (1) the mean species length calculated from all length measurements recorded within the same sub‐feature habitat type in this study; (2) if no proxy lengths for that habitat were available, then the species mean from the entire study was used; and (3) if no recorded lengths from the entire study were available for a species, then a proxy mean length was sourced from FishBase. For individuals only identified to a family level, the study level family mean length was used.

To visualize the spatial distribution of species richness, relative abundance, diversity, and biomass, a 1‐km^2^ hexagonal grid was generated using the Terra package in R (Hijmans et al., 2024). Deployments were spatially intersected with this hexagonal grid, and the mean value for each index was aggregated for all deployments within each cell. To further understand the variation in demersal species richness, relative abundance, and total biomass within the archipelago, we tested for differences between designated feature habitat types. Differences between the main habitat types (Reefs and sediments) were tested using a nonparametric Mann–Whitney *U* test, with sub‐habitat tested using a post hoc Kruskal–Wallis *H* test.

### Drivers of species richness, relative abundance, and biomass

In addition to habitat type, seven predictor variables (Appendix S1: Table S1) were identified as potential drivers of richness, relative abundance, and biomass. Depth (in meters) and sea surface temperature (SST, in degrees Celsius) were recorded in situ using the vessel‐mounted Garmin transducer (model GT54UHT) for each deployment location. A relief score was visually assessed to estimate benthic topographic complexity (Lester et al., 2022), scaled 0 (no vertical relief) to 5 (exceptionally complex with numerous caves and overhangs) according to Wilson et al. (2007). The wider seabed variability surrounding the BRUV deployments was quantified using the mean slope gradient (° measured in degrees) and the range of seabed ruggedness (in meters). These variables were calculated using the “Raster terrain analysis” plugin in QGIS (v3.34.3), GEBCO Gridded Bathymetry Data and a 200‐m bounding buffer from each deployment. Given the oceanic location of the archipelago, an exposure metric (° measured in cardinal degrees) was calculated to explore the influence of the prevailing Westerly currents and winds on marine assemblages (Brown et al., 2022; Friedlander et al., 2003). Exposure was estimated by taking the mean bathymetric aspect (“Raster terrain analysis”—“Aspect”) from the 200‐m buffer zone surrounding a deployment and calculating the number of degrees from due west (270°), the direction of prevailing currents and winds locally (Exeter et al., 2024), with those farthest away having the lowest exposure values (0–180°). Finally, a remoteness measure (in meters) was calculated as a deployment's minimum distance from an inhabited island (Figure 1) as a proxy for cumulative human impacts (i.e., vessel noise pollution and disturbance, point source pollution and fishing accessibility).

To test for multicollinearity between predictor variables prior to model fitting (Dormann et al., 2013), Pearson's coefficient of correlation and a variance inflation factor (VIF) test were applied. All variables were found to be weak or moderately correlated (correlation coefficient all <0.55 with a VIF value below 5), except for ruggedness paired with relief score and mean slope paired with remoteness, ruggedness, and relief score (Appendix S1: Tables S2 and S3). We therefore chose to omit both ruggedness and mean slope from further analyses, leaving five predictor variables of interest (Appendix S1: Table S1) in addition to habitat type.

To explore the potential drivers of ecological indices derived from the BRUV data, we ran a series of generalized linear models (GLMs), using an information theory approach to allow us to compare non‐nested models (Harrison et al., 2018). We built a candidate model set based on a priori hypotheses (*n* = 17 models per variable). Our most complex model contained effects of depth and relief score (Appendix S1: Table S4). We also fitted models containing single predictors and with habitat type (reef or sediment) as an interaction term (Appendix S1: Table S4). While preliminary analysis of the data suggested that there were no significant effects of year in the data (nonparametric Mann–Whitney *U* test; demersal richness *p* = 0.14, *W* = 10,781; demersal abundance *p* = 0.11, *W* = 10,858; total biomass *p* = 0.07, *W* = 8549; Appendix S1: Figure S2), we chose to include the year in all models to control for sampling occurring over two consecutive seasons. Despite the nonsignificant effect of year were removed from total biomass models due to the presence of dense shoals of Atlantic horse mackerel (*Trachurus trachurus*) and large‐bodied predatory fish (Atlantic bluefin tuna (*Thunnus thynnus*)) in several 2023 deployments, resulting in multiple extreme outlier data points. Re‐running the Mann–Whitney *U* test without Seven Stone's deployments resulted in a nonsignificant difference for total biomass (*p* = 0.18, *W* = 7942; Appendix S1: Figure S2).

Species richness was modeled using a Poisson error distribution as the models showed no evidence of overdispersion (mean = 5.4, variance = 4.9, Dispersion Index = 0.91). We used a negative binomial distribution for relative abundance (summed MaxN values per deployment; mean = 24.1, variance = 2406.5, Dispersion Index = 99.9) and a gamma distribution for total biomass (mean = 6.1, variance = 36.0, Dispersion Index = 6.0), all with a log link function, to account for overdispersion identified in exploratory data analysis. Due to low level but significant spatial autocorrelation (Moran's *I* test, *p* < 0.05) detected in model residuals during preliminary testing, a spatially lagged dependent variable (Dormann et al., 2007) weighted by the four closest neighboring deployment locations was included in all models as a predictor using the spdep R package (Bivand et al., 2024). Generalized linear mixed models with site coordinates (Lat, Lon) included as a random effect using the “fitme” function in the spaMM R package (Rousset et al., 2024) were also explored; however, they failed to adequately resolve spatial autocorrelation in model residuals (Appendix S1: Table S5). Models were ranked according to sample size‐corrected Akaike information criterion (AIC_c_) values, with all models within two units of AIC_c_ of the top performing model retained.

### Drivers of indicator taxa body length

To explore the drivers of body length, a subset of indicator taxa was selected based on their detectability using BRUV systems and their commercial (i.e., likely to experience pressure from local or external fisheries) or conservation importance (i.e., a designated species feature of conservation interest, or listed as threatened in a global conservation index such as IUCN red list). This indicator list comprised seven species: *Scyliorhinus canicula* (small‐spotted catsharks; commercial external), *Scyliorhinus stellaris* (nursehound; commercial external and conservation), *Labrus bergylta* (ballan wrasse; commercial external), *Labrus mixtus* (cuckoo wrasse; commercial external), *Pollachius pollachius* (European pollack; commercial external), *Cancer pagurus* (brown crab; commercial local, external), and *Palinurus elephas* (European spiny lobster; commercial local, external, and conservation). Body length was modeled using GLMs with a Gaussian link function against the same ranking criteria and predictor variables outlined above.

### Species rarefaction

To examine the effectiveness of the survey sampling effort at capturing species present within the archipelago, rarefaction curves (Colwell et al., 2012) were generated using the iNext R package (Chao et al., 2014). Rarefaction curves provide a statistical expectation of species accumulation against sampling effort and were calculated for all data aggregated and for the main feature habitats of the SAC (reefs and subtidal sediments) with extrapolations based on 1500 samples equivalent to a 5.4‐fold increase in existing deployments.

## RESULTS

Benthic BRUV systems were deployed as close as possible to the GRTS‐generated coordinates but were adjusted where necessary due to obstructions (i.e., submerged rocks) or vessel transit routes. If relocated, deployments were still spaced >200 m apart (mean = 361 m), with only three relocated deployments found to be <200 m (min = 152 m) on the same sampling day. Of the 300 GRTS‐generated deployment locations, 280 (39 mono, 241 stereo) were retained for further analysis. Of these, 28 had to be resampled due to obstructions (*n* = 17), relocation being too close to another sample (*n* = 3), orientation of BRUV units (*n* = 5), bait cage failure (*n* = 2), and camera failure (*n* = 1). For the remaining 20 locations, poor weather prevented resampling of 10 obstructed deployments, and 10 deployments were deemed unsafe due to underwater hazards with no viable relocation sites within the vicinity. Successful deployments were collected across 54 days, with 135 deployments in 2023 and 145 in 2024 (Appendix S1: Table S6). Upon visual inspection of footage, 181 deployments were within Reef‐dominated habitats (circalittoral rock *n* = 82, infralittoral rock *n* = 99), and 99 within Subtidal sediment habitats (Sediments *n* = 75, Subtidal seagrass beds *n* = 24). The dynamic and patchy nature of seagrass beds resulted in 19 of the 50 GRTS locations being found to be sediment dominated in situ. Fifteen of the 100 circalittoral reef deployments were found to be macroalgae‐dominated infralittoral reef, with kelp‐dominated reef recorded as deep as 26.2 m, reflecting the low turbidity and high water clarity across the archipelago (Warwick et al., 2003).

### Species observations

**FIGURE 2:**
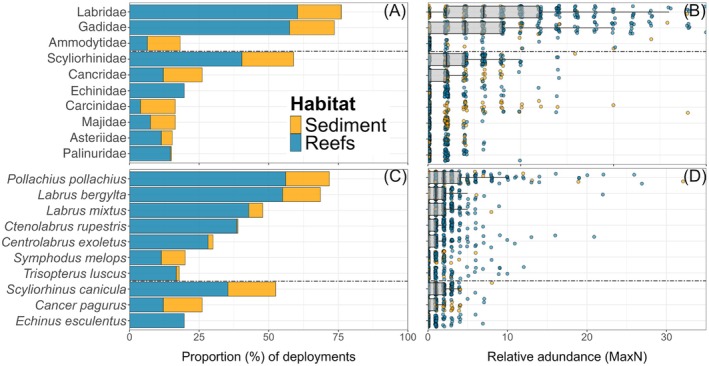
Proportion of total deployments observed and relative abundance of the top 10 most commonly occurring families (A, B) and species (C, D). Note, Relative abundance axis capped at MaxN 15 for clarity, but *Ammodytidae* spp. and *Pollachius pollachius* include observed values >15.

From the 280 deployments, a total of 11,950 individuals were recorded (summed MaxN values), representing 64 species and 44 families (Appendix S1: Table S7). Of these, 6743 individuals were demersal‐associated fish, elasmobranchs, or invertebrates, representing 51 species and 32 families. Teleost fish were the most dominant group in terms of species richness (*n* = 41), with 9 crustaceans, 5 other benthic invertebrate species, 3 elasmobranch species, 2 cephalopods, 2 species of seabird, and 2 marine mammal species recorded. The most abundant families were Ammodytidae (mean ± SD MaxN across all deployments: 11.7 ± 48.0), Carangidae (4.6 ± 53.8), and Labridae (4.5 ± 6.0) (Figure 2). Labridae were the most commonly occurring, recorded across 76.4% of all deployments, followed by Gadidae (73.9%) and Scyliorhinidae (58.9%; Figure 2). *Ammodytes tobianus* (lesser sand eel) were the most abundant species (mean ± SD maxN: 11.7 ± 48.0) but were predominantly found in rare hyperabundant schools and only occurred in 17.5% of deployments. In comparison, *Pollachius pollachius* were the most commonly observed species (72.1% of deployments), followed by *Labrus bergylta* (68.9%) and *Scyliorhinus canicula* (52.5%). *P. pollachius* were also the most abundant demersal‐associated species (mean ± SD maxN: 3.3 ± 5.1), with *S. canicula* the most abundant elasmobranch (1.0 ± 1.2) and *Cancer pagurus* the most abundant crustacean (0.46 ± 1.0).

**FIGURE 3:**
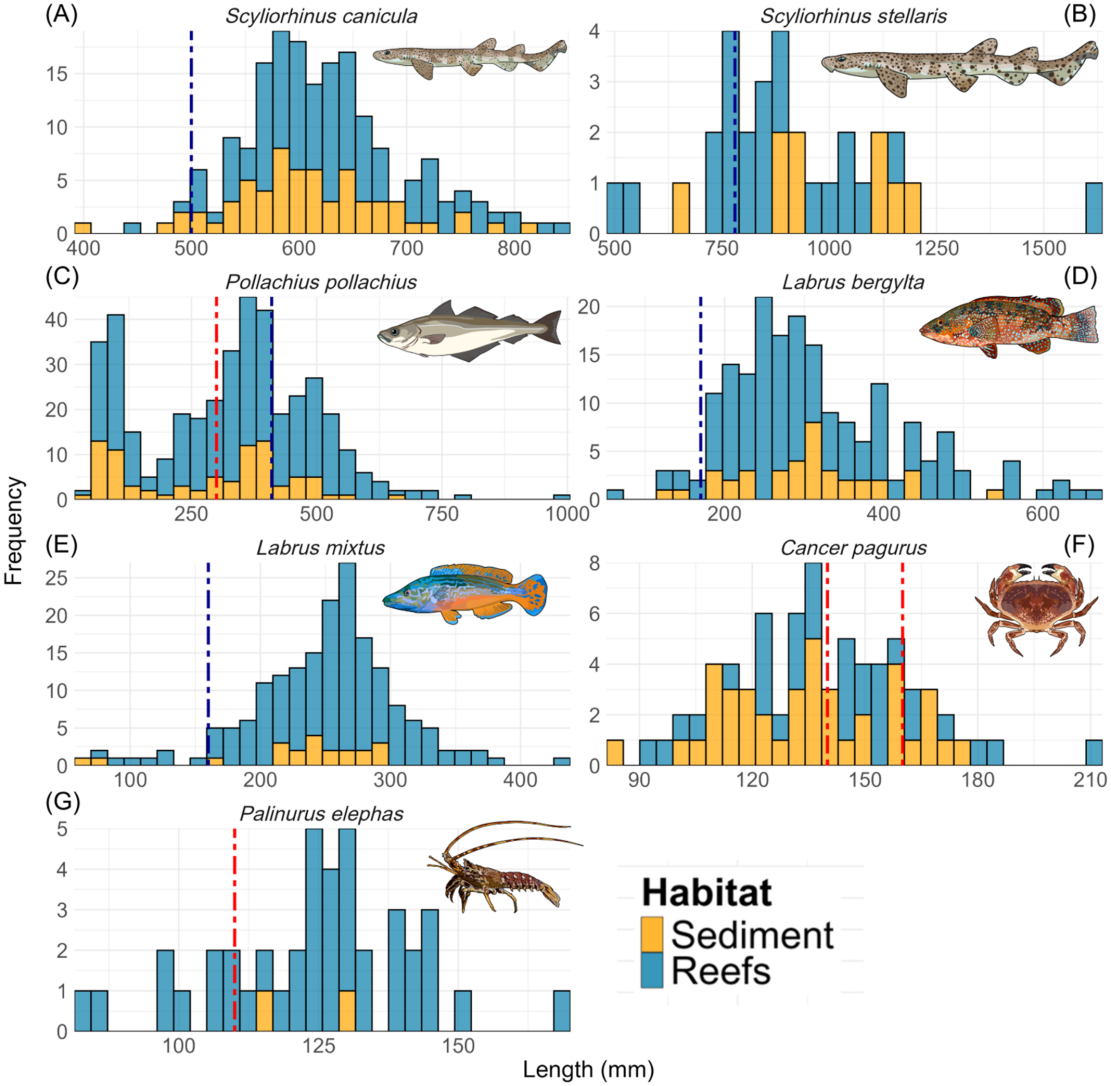
Length frequencies for indicator taxa of interest. Red dashed line indicates commercial or recreational minimum landing sizes within the Isles of Scilly six nautical mile inshore fisheries zone (note both male and female minimum landing size shown for *C. pagurus*). Blue dashed lines indicate length of maturity sourced from FishBase or Marlin (www.marlin.ac.uk) if not available. Species illustrations by Emma Wood.

Length measurements were possible for 43 of the 64 species recorded, with a total of 1641 individuals measured. The mean length recorded for all species was 299 mm (±210 mm), with the largest individual being a *Thunnus thynnus* (Atlantic bluefin tuna—1895 mm) and the smallest a *Centrolabrus exoletus* (rock cook—44 mm). *P. pollachius* was the most measured species (*n* = 403), followed by *L. bergylta* (*n* = 189) and *Labrus mixtus* and *S. canicula* (both *n* = 182). For commercially targeted species within the archipelago, the majority (82.9%) of *Palinurus elephas* were larger than the local MLS of 110 mm or the national MLS as of 2023 of 90 mm (Figure 3). *C. pagurus* was found to be mostly undersized compared to the commercial MLS. The length frequency for *P. pollachius*, a species heavily targeted by commercial vessels both inshore and outside the 6 nautical mile fisheries zone, appeared to be bimodal, with a peak for both juveniles (55–120 mm) and legal, but sub‐adult individuals (300–410 mm). Both catshark species (*S. canicula* and *S. stellaris*) were found to be comprised of individuals larger than the size of maturity (Figure 3). The majority of measurements (51.4%) used to inform biomass calculations were true values or used a species‐specific measurement from the same BRUV sample, as opposed to using a proxy value (Appendix S1: Table S8). Sub‐feature habitat proxy measurements were used for 38.7% of species biomass calculations, with study level, FishBase, or family‐level proxies all contributing less than 10% of measurements to biomass calculations.

### Species richness, abundance, diversity, and biomass

**FIGURE 4:**
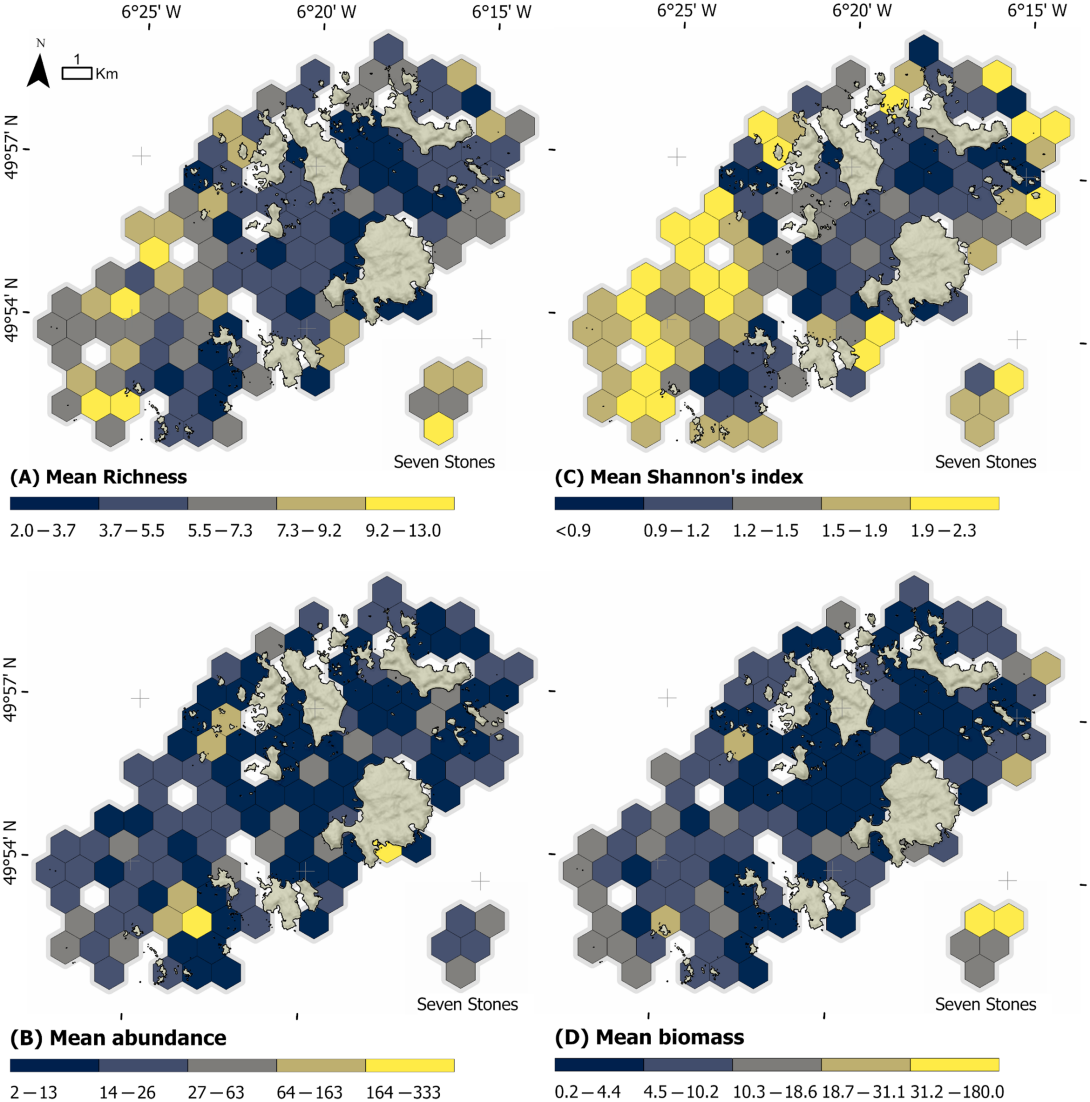
Mean values for (A) demersal richness, (B) demersal relative abundance, (C) Shannon–Wiener Diversity Index, and (D) total biomass for all deployments at a 1 km^2^ hexagonal grid cell resolution. All intervals displayed using a Natural Breaks (Jenks) function. Maps generated in ArcPro (v3.1) and Mapbox.

Spatial trends among all BRUV deployments suggest areas of elevated demersal richness and diversity in the reefs and deeper sediments habitats in the west of the archipelago and at the remote Seven Stones Reef (Figure 4). Spatial trends in demersal abundance and total biomass were harder to determine; however, localized hotspots were again evident to the west and around the Seven Stones Reef, albeit to a lesser extent (Figure 4).

**FIGURE 5:**
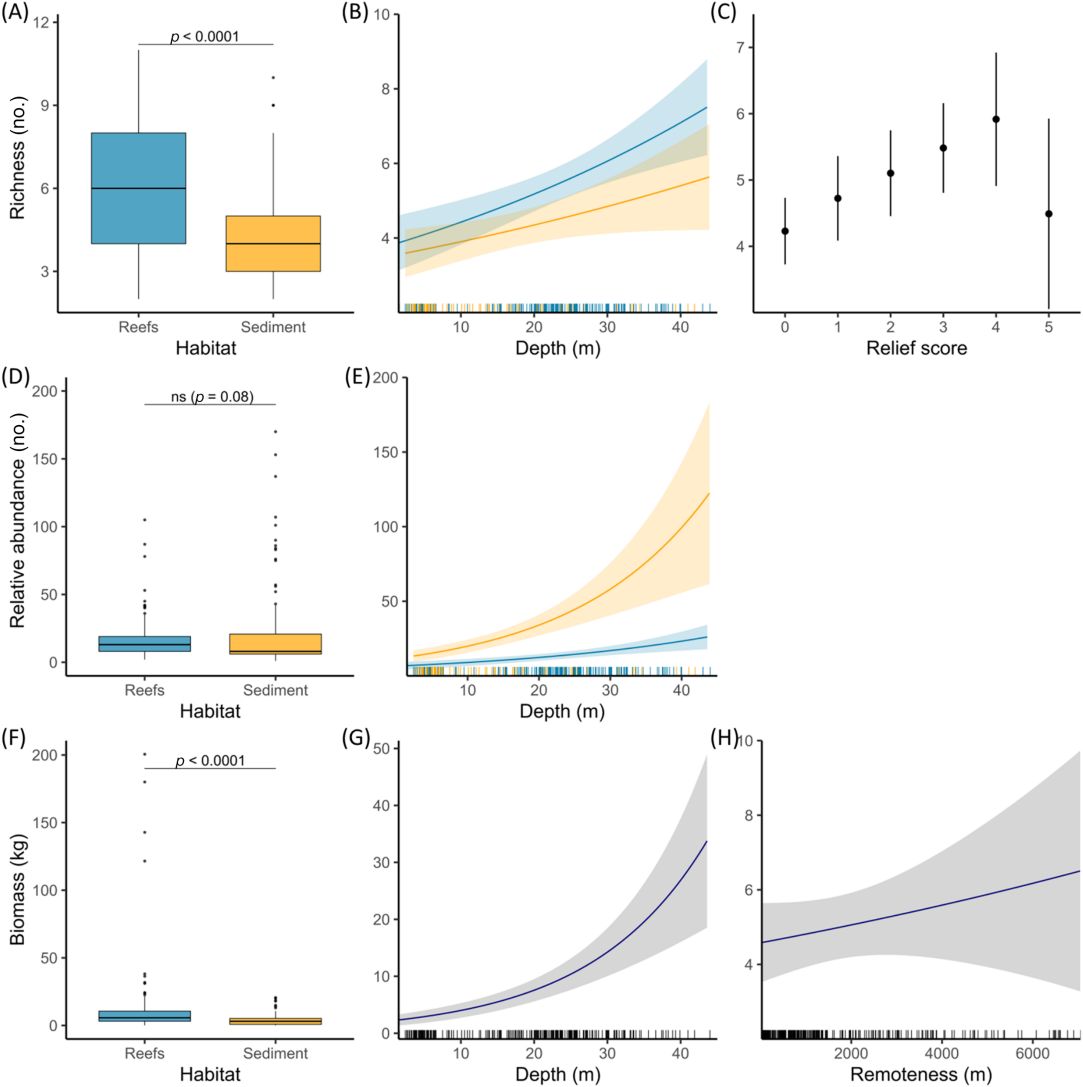
Interhabitat comparisons (box and whiskers plot with results of Mann–Whitney *U* test shown) and predicted values from generalized linear models representing the effect of environmental and physical variables from the highest‐ranked (within two units of corrected Akaike information criterion) models on demersal species richness (A–C), relative abundance (D, E) and total biomass (F–H).

Reef and subtidal sediment habitats exhibited significantly different levels of richness for demersal species (Mann–Whitney *U* test, *p* < 0.001, *W* = 13,144) with higher richness observed within reef habitats (mean ± SD: 6.0 ± 2.1) than in sediments (4.3 ± 1.9; Figure 5A). Kruskal–Wallis and Dunn's pairwise post hoc tests (Bonferroni adjustment) revealed significant differences in richness between certain sub‐habitat types, with circalittoral reefs distinct from all other sub‐habitats (*p* < 0.001), and infralittoral rock significantly different from subtidal sediment habitats (*p* = 0.01; Appendix S1: Figure S1). However, infralittoral rock and subtidal seagrass (*p* = 0.35) and subtidal seagrass and subtidal sediments (*p* = 0.44) displayed no significant differences. The highest richness was observed on circalittoral reefs (mean ± SD: 7.3 ± 1.9), followed by infralittoral rock and subtidal seagrass, with the lowest richness observed in bare sediment habitats (4.2 ± 2.0) (Table 1). Shannon index scores displayed similar trends to richness, with reefs displaying significantly (*p* < 0.001, *W* = 13,642) higher diversity scores (1.5 ± 0.5) than sediment habitats (1.0 ± 0.5), notably within circalittoral reef habitat (1.76 ± 0.43; Table 1).

**TABLE 1:** Mean and SD of demersal species richness, relative abundance (MaxN), and total biomass aggregated by main and sub‐habitat type.

Habitat	Sub‐habitat	Richness (no.)		Relative abundance (no.)		Biomass (kg)		Diversity	
		Mean	SD	Mean	SD	Mean	SD	Mean	SD
Reefs		6.04	2.13	15.8	13.4	10.94	24.07	1.50	0.50
	Circalittoral	7.33	1.87	19.8	13.9	15.5	31.15	1.76	0.43
	Infralittoral	4.97	1.7	12.5	12	7.17	15.16	1.29	0.45
Sediments		4.25	1.9	39.2	78.5	4.16	4.57	1.00	0.50
	Seagrass	4.42	1.61	30.2	38.3	2.64	2.75	0.96	0.39
	Sediments	4.2	1.99	42.1	87.6	4.64	4.93	1.02	0.53
Total		5.4	2.22	24.1	49.1	8.54	19.8	1.32	0.55

In comparison, no significant differences between reef and sediment habitats were found for the relative abundance of demersal species (Mann–Whitney *U* test, *p* = 0.08, *W* = 10,075), despite sediments having higher mean relative abundance values (15.8 ± 13.4 and 39.2 ± 78.5, respectively; Figure 5). Significant differences were found between sub‐habitat types (Kruskal–Wallis, *p* < 0.001), but post hoc tests revealed only circalittoral reefs to be significantly different from infralittoral rock and subtidal sediments (both *p* < 0.001), with all other pairwise tests found to be nonsignificant (*p* > 0.05; Appendix S1: Figure S1). Relative abundance was found to be highest within subtidal sediment habitats (mean ± SD: 42.1 ± 2.1), likely driven by Ammodytidae shoals, with the lowest observed in infralittoral rocky habitat (12.5 ± 12.0). As with richness, total biomass was found to be significantly higher in reef habitat (Mann–Whitney *U* test, *p* < 0.001, *W* = 12,435), with circalittoral reefs again found to be significantly distinct from all other sub‐habitat types by the post hoc tests, with the highest biomass levels (15.5 ± 31.2 kg, Table 1). Seagrass was found to have the lowest mean biomass (2.6 ± 2.8 kg), despite having the second highest relative abundance (30.2 ± 38.3).

### Drivers of species richness, relative abundance, and biomass

The best‐supported GLM model for trait richness contained effects of relief score and depth, which explained 39.0% of variation (pseudo *R* ^2^), with depth and habitat type as an interaction term within two AIC_c_ units and explaining 36.5% of the variation (Table 2). Demersal species richness increased with depth and with increases of relief score, with the exception of the highest relief category (Figure 5). For relative abundance, the best‐supported model included depth with habitat type as an interaction term, which explained 33.9% of the variation (Table 2). Relative abundance was found to increase with depth; however, increases were driven by subtidal sediment habitats, with only a limited increase in relative abundance with increased depth within reef habitats (Figure 5). The best‐supported model for total biomass (in kilograms) included the depth predictor variable, both with and without habitat type as an interaction term, with depth and remoteness also within two units of AIC_c_ (Appendix S1: Table S4). Elevated biomass was found to increase with depth. Depth, however, only explained 22.9% of the variation in biomass. Total biomass was the only ecological response variable to include remoteness, acting as a proxy for human pressures, in one of the best‐fitting models (Figure 5). SST was not included in any of the best‐fitting models for either richness, relative abundance, or biomass. Similarly, despite the oceanic location of the archipelago, a model containing exposure as a single term had weaker support compared to the top model (Delta AIC) for richness (ΔAIC_c_ = 27.17), abundance (ΔAIC_c_ = 79.69) or biomass (ΔAIC_c_ = 33.45; Appendix S1: Table S4). For all models using the spatially lagged response predictor, no significant spatial autocorrelation (Moran's *I* statistic, *p* < 0.05) was found in model residuals for any of the best‐fitting models (Appendix S1: Table S9).

**TABLE 2:** Generalized linear model (GLM) results of the highest‐ranked models within two units of corrected Akaike information criterion (AIC_c_) for predicting demersal species richness, relative abundance, and total species biomass.

Index	Model terms^a^	AIC_c_	Weight	Pseudo *R* ^2^	ΔAIC_c_ to null model
Richness	Relief score + Depth	1149.1	0.47	0.390	85.67
	Depth × Habitat	1149.3	0.42	0.365	
Relative abundance	Depth × Habitat	2240.5	0.98	0.339	116.05
Total biomass	Depth	1417.9	0.40	0.229	73.80
	Depth + Remoteness	1418.5	0.29	0.233	
	Depth × Habitat	1419.0	0.23	0.237	

^a^ All models include the spatially lagged term and year effect.

### Drivers of indicator taxa body length

**FIGURE 6:**
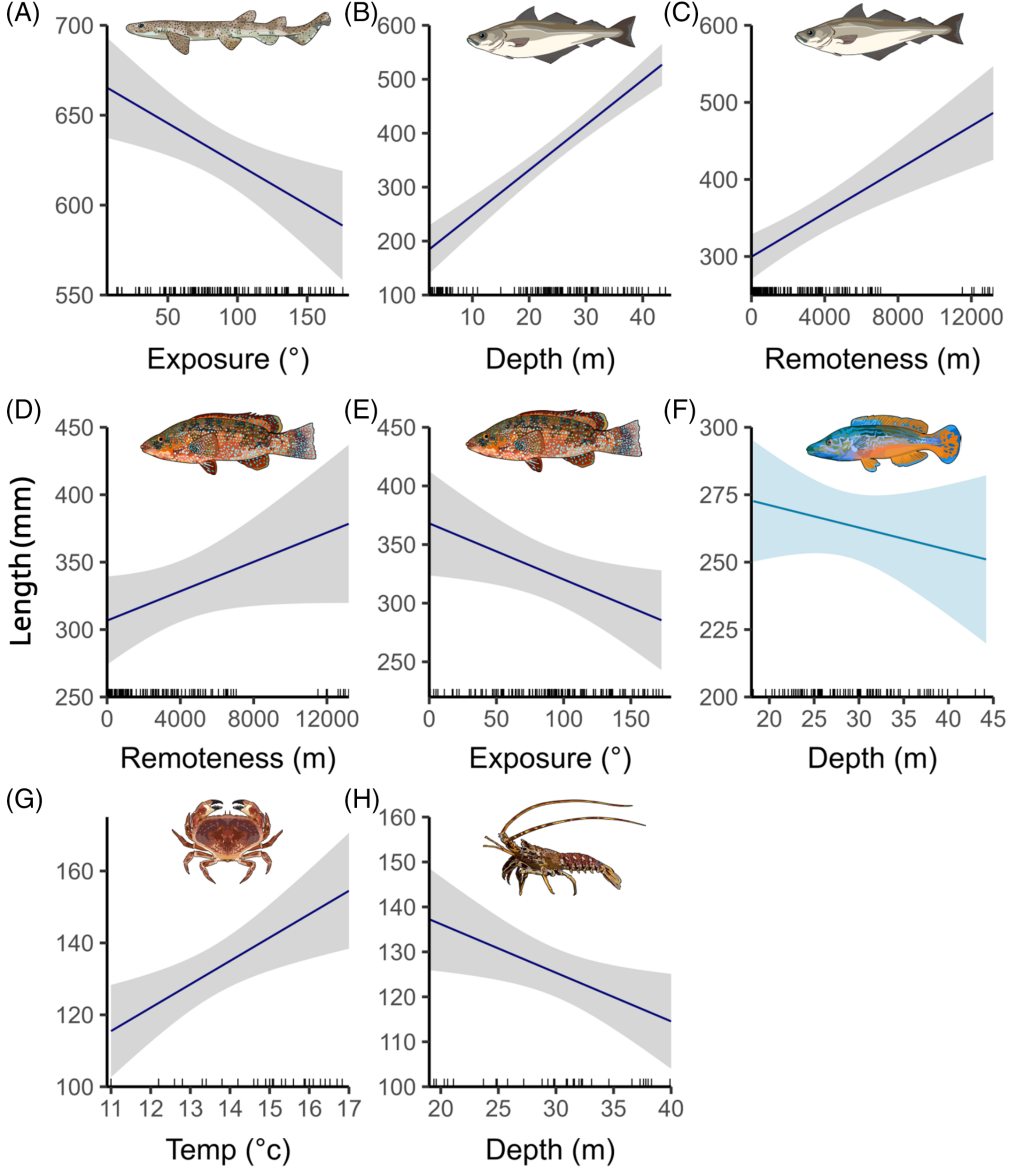
Predicted values from generalized linear models representing the effect of environmental and physical variables from the highest‐ranked (corrected Akaike information criterion) models on indicator taxa body length (in millimeters) for (A) *Scyliorhinus canicula—*small‐spotted catshark; (B, C) *Pollachius pollachius—*European pollack; (D, E) *Labrus bergylta—*ballan wrasse; (F) *Labrus mixtus—*cuckoo wrasse (reef deployments only); (G) *Cancer pagurus—*brown crab and (H) *Palinurus elephas—*spiny lobster. Species illustrations by Emma Wood.

A variety of predictor variables were found to drive body size for indicator taxa, with variables such as depth having contrasting interspecific effects. The best‐performing model for *S. canicula* was exposure, with larger individuals predicted to occupy more sheltered sites (Figure 6A, Appendix S1: Table S10A). Models including terms for remoteness, exposure, and an exposure: habitat type interaction were also within two units of AIC_c_ (Table 3). Depth and remoteness were the best predictors of *P. pollachius* body length (Figure 6B,C), with larger individuals found in deeper waters and in areas further from inhabited islands. *L. bergylta* body size was predicted to increase in more remote deployments that were less exposed (Figure 6D,E). Depth was also the best‐performing predictor of *L. mixtus* body length, with larger individuals predicted to occupy shallower deployments (Figure 6F). For commercially exploited crustacean species, *C. pagurus* carapace width was positively associated with increased SST, suggesting that either a seasonal effect or areas of warmer water within the archipelago held larger individuals (Figure 6G). *P. elephas* carapace length was found to decrease with depth (Figure 6H), albeit based on a limited sample size (no. deployments = 31, no. lengths = 41) and with the null model within two units of AIC_c_. No models were found to perform better than the null model for *S. stellaris* body length, likely due to the smaller sample size (no. deployments = 27, no. lengths = 31) and size distribution appearing to span a variety of habitats and environmental gradients.

**TABLE 3:** Generalized linear model (GLM) results of the highest‐ranked (corrected Akaike information criterion [AIC_c_]) models for predicting indicator species body length.

Species	Model terms	AIC_c_	Weight	Pseudo *R* ^2^	ΔAIC_c_ to null model
*Scyliorhinus canicula*	Exposure	1254.5	0.4	0.10	7.37
*Pollachius pollachius*	Depth + Remoteness	1562.5	1	0.70	151.3
*Labrus bergylta*	Remoteness + Exposure	1406.4	0.35	0.098	5.65
*Labrus mixtus*	Depth × Habitat	1001	0.99	0.257	19.09
*Cancer pagurus*	Temp	401.7	0.80	0.22	6.63
*Palinurus elephas*	Depth	264.2	0.4	0.173	0.77

### Species rarefaction

**FIGURE 7:**
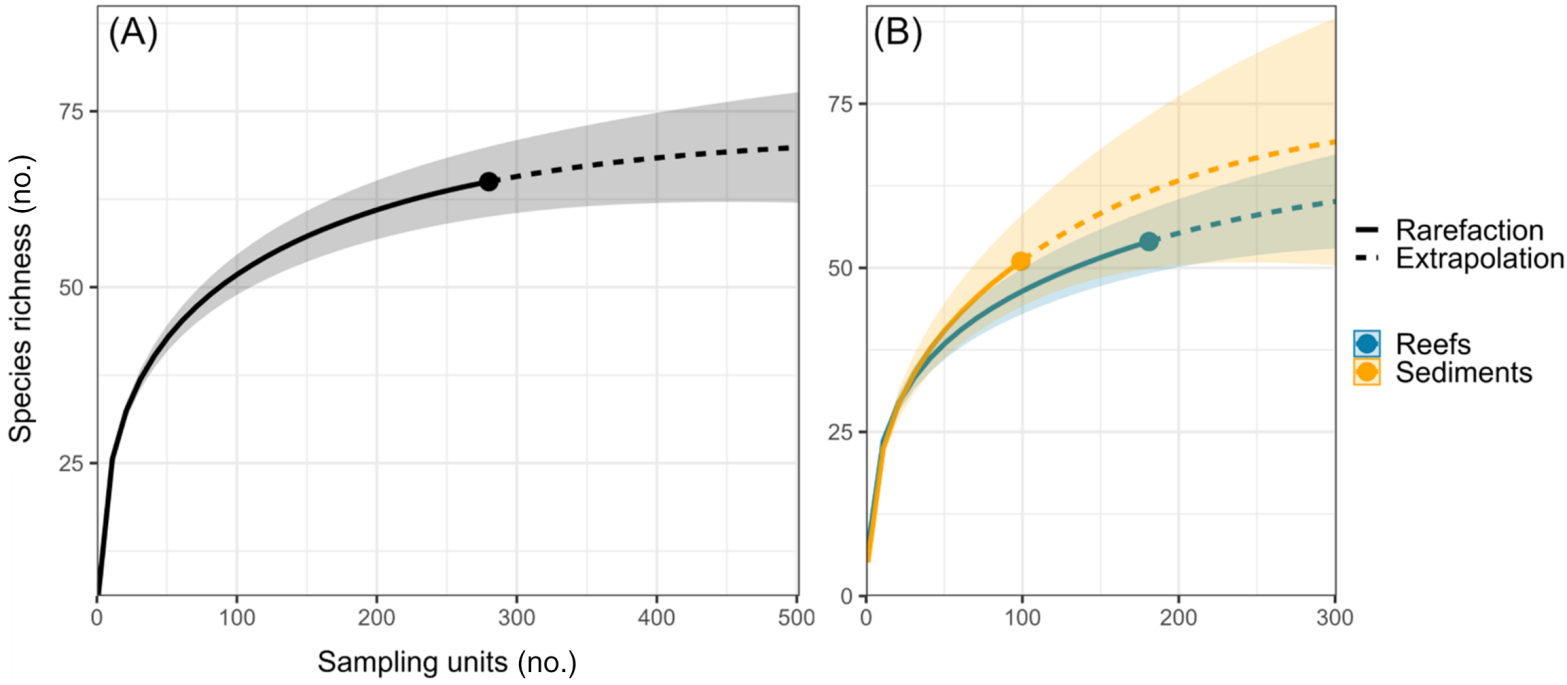
Rarefaction (solid line) and extrapolation (dashed line) of species richness from 280 baited remote underwater video deployments, with a 95% unconditional CI shown (shaded area). Rarefaction conducted using all samples aggregated (A) and for each Special Area of Conservation feature habitat of interest separately (B).

Rarefaction curves generated from the BRUV dataset suggest that sampling effort was largely effective at capturing the species present within the study area. An additional 601 hours of BRUV data were estimated to record just 6 new species (based on an extrapolated asymptote of 71.1 species [95% CI = 60.6–81.5]) for the complete dataset (Figure 7). However, extrapolation based exclusively on subtidal sediment data estimated an additional 6 species in just 42 h of effort (asymptote 57.2 species, 95% CI = 47.9–66.5), equivalent to ~14 field days, suggesting that further samples within sediment habitats may be beneficial.

## DISCUSSION

Despite being recognized as a regionally important marine biodiversity hotspot (Lewis et al., 2008; Warwick et al., 2003), the Isles of Scilly archipelago exemplifies a system with limited data on many of its marine assemblages (Exeter et al., 2024). This study provides a contemporary inventory of mobile marine species, their abundance, and biomass, and identifies key areas of elevated biodiversity within the archipelago's waters and MPAs. We also demonstrate the effectiveness of stereo BRUV systems as a flexible and low‐cost tool for sampling in UK waters. To our knowledge, this study represents the largest stereo BRUV survey ever conducted in UK waters (Hall et al., 2021; Peters et al., 2015; Unsworth et al., 2014), and the most spatially comprehensive BRUV survey of a single UK site. Results can therefore guide and inform a variety of local management interventions discussed below and inform the wider application of stereo BRUV as a fisheries‐independent survey tool in the UK MPA network and marine systems of the North East Atlantic more broadly.

### Species richness, abundance, diversity, and biomass

The Isles of Scilly were found to support high levels of species richness and relative abundance when compared to the limited number of published BRUV studies regionally. Of the only study with a comparable sample size, from the North coast of Cornwall, similar levels of species richness were recorded (*n* = 67 species from 46 families; Bicknell et al., 2019), as were the maximum number of species identified in a single sample, with max richness of 13 species recorded in this study compared to 12 in Cornwall. Relative abundance of teleost fish was estimated to be higher in the Isles of Scilly (mean ± SD: 24.1 ± 49.1) than at North Cornish sites (Bicknell et al., 2019). The Isles of Scilly also displayed higher mean richness (mean ± SD: 5.5 ± 2.3) and abundance (mean ± SD: 32.7 ± 94.3) when considering only mobile species (i.e., excluding small demersal invertebrates) than a comparable MPA in the UK Channel Islands (richness mean ± SD: 4.9 ± 1.8; MaxN mean ± SD: 23.8 ± 15.2; Blampied et al., 2022). Results also indicate the archipelago supports high abundances of commercially important and conservation‐concern species such as *P. elephas* (mean ± SD MaxN: 0.20 ± 0.56), *P. pollachius* (3.3 ± 5.1), and the IUCN Red‐listed *S. stellaris* (Vul. [Finucci et al., 2021]) (0.25 ± 0.61) relative to other regional studies where reported MaxN values were considerably lower (i.e., Blampied et al., 2022; Unsworth et al., 2014). In contrast, other gadoid species (e.g., *Trisopterus minutus*) appeared to occur at lower abundance levels compared to mainland sites in the Southwest of the UK (i.e., Davies et al., 2021). The diversity (number of species = 6) and abundance (4.38 ± 4.88) of Labridae species were also found to be high (Figure 2), relative to regional studies with reported values (Blampied et al., 2022; Unsworth et al., 2014) and may be indicative of healthy reef and seagrass systems (Bourlat et al., 2021). These results may also reflect lower levels of anthropogenic pressures compared to mainland systems, such as Cornwall, where there is a live capture wrasse fishery (Henly et al., 2021).

While data gaps regarding the marine assemblages of the Isles of Scilly motivated this study, anecdotal and historical ecological records can provide ad hoc insights into climate‐driven regime shifts or local species declines (Herdson, 2010). For instance, the more northerly *Eledone cirrhosa* (Curled octopus), which was reported to persist in the archipelago until recently (Herdson, 2010), was not recorded, whereas the warmer water *Octopus vulgaris* (Common octopus) was observed, though at very low levels. Notably, there was an absence of *Rajidae* (skate) species and a low abundance of flatfish, despite species such as juvenile *Pleuronectes platessa* (European plaice) and *Raja clavata* (thornback ray) being described as historically abundant in the archipelago (Herdson, 2010). While BRUV systems have successfully recorded skate species regionally (Blampied et al., 2022), detection rates for skates and batoid species are generally lower than for benthic sharks when using BRUV systems, particularly in sediment‐based habitats (McIvor et al., 2022; White et al., 2013). It is therefore difficult to determine whether results indicate local population declines or reflect biases in BRUV surveys for sampling *Rajidae* spp. Complementary methods, such as eDNA or in water surveys, could therefore be beneficial to validate the findings of this study, and detect cryptic or rare species in future surveys (Clark et al., 2024). The advent of metaprobes has improved the accessibility of eDNA sampling as a complementary noninvasive technique (Maiello et al., 2022) that can help validate BRUV observation, and help detect rare or cryptic species (i.e., Clark et al., 2024).

High detection rates of *P. elephas* (42.7% of circalittoral reef deployments) support findings from fisheries‐dependent data, indicating that the species is in a recovery phase after decades of population decline (Jackson, 2021; Morcom et al., 2023). These results suggest that BRUV surveys can serve as a complementary method to established traditional dive surveys, such as Seasearch (Jackson, 2021), specifically monitoring datasets for *P. elephas* in MCZ sites where it is a designated feature (Natural England, 2019). The ability to measure individuals using stereo BRUV technology is particularly valuable for tracking trends in age classes and stock biomass (Galaiduk et al., 2017). Analysis of *P. elephas* length data from this study suggests that BRUV systems may be biased toward larger individuals (mean ± SD: 124.5 ± 17.5 mm, range: 82.3–168.0 mm, Figure 3G) compared to at‐sea observer sampling from the same period which detected smaller individuals landed in trammel (tangle) nets (mean: 112 mm) (Morcom et al., 2023). This skew could be due to the more cryptic and nocturnal behavior of juvenile spiny lobsters, which may be less likely to leave refuges during the day (Withy‐Allen & Hovel, 2013), or due to the limited length sample size in this study. The high number of larger individuals recorded in the BRUV data may also reflect the placement of BRUV systems outside areas of high fishing pressure, or the influence of bait compared to the passive nature of static nets. BRUV deployments in this study may therefore include specific reef sites that experience limited fishing pressure and hold a larger size class of *P. elephas*. In contrast, fisheries‐dependent landing data are likely influenced by repeated fishing pressure, which tends to remove larger individuals from the population (Hsieh et al., 2010). Dedicated use of stereo BRUV systems in *P. elephas* core habitats could therefore enhance the management of the species, particularly within MPAs (Stobart et al., 2015), with trials of nighttime deployments to detect smaller individuals being warranted. Additional research into the viability of BRUV data for sexing individuals could also be beneficial, given that only ~10%–12% of individuals in landings data are female (Morcom et al., 2023).

### Drivers and distribution of biodiversity

Spatial visualization and modeling reveal elevated richness, diversity, and biomass in deeper reef habitat, notably deployments with higher benthic complexity (relief) toward the west of the archipelago (Figure 4). These variables also drove larger body size in important commercial species (Galaiduk et al., 2017), such as *P. pollachius* (European Pollack) that has seen a zero Total Allowable Catch quota implemented in 2024. The ecological importance of these sites has implications for local monitoring and management. While byelaws covering much of the SAC and most MCZ‐designated MPAs currently exclude bottom‐towed fishing gears from these zones (Exeter et al., 2024), shifts in fishing practices to target *P. elephas* have resulted in a transition from low‐impact potting (Davies et al., 2021) to tangle nets, notably in areas overlapping these diverse western reef sites (Morcom et al., 2023). Tangle (trammel) nets have lower selectivity than potting and are associated with high bycatch rates of nontarget species (Amengual‐Ramis et al., 2016) including a variety of elasmobranch species (Marine Institute and Mhara, 2023). Long‐term monitoring within these ecologically important zones, notably for species such as *S. stellaris*, will therefore be essential for monitoring the ecological health of the SAC and fisheries impacts on these reefs and their communities (English Nature, 2000). While local MCZs (Figure 1) were designated for Features of Conservation Interest (FOCI; JNCC, 2016), typically sessile species unsuited to BRUV surveying (with the main exception being *P. elephas*), most sites overlapped with medium to high demersal species richness (Appendix S1: Figure S3). The very highest levels of richness were generally found to fall outside the existing MCZ network, however (Appendix S1: Figure S3), suggesting either a low correlation between mobile and sessile species of conservation importance or reflecting data gaps in the original MCZ designation process (Lieberknecht et al., 2011).

The Seven Stones Reef, which is not fully protected and was the site of the 1967 Torrey Canyon oil spill, considered one of the worst maritime ecological disasters in European waters (Wells, 1970), was also found to be a hotspot of biodiversity. The Reef system displayed higher richness (mean ± SD: 7.73 ± 1.75), biomass (52.5 ± 70.2), and diversity (1.46 ± 0.67), and similar relative abundance (23.5 ± 11.3) when compared with deployments within the main archipelago (Table 1). Several distinct species not observed across other deployments were also recorded within this reef system. This included pelagic and deepwater species such as *T. thynnus* (Atlantic bluefin tuna), *Capros aper* (boarfish), and *Acantholabrus palloni* (scale‐rayed wrasse), which have only been observed in the Southwest UK on eight previous occasions (Hiscock & Earll, 2024). The isolated nature of the Reef's pinnacle system may be acting as an important aggregation or migration site for mobile species in the region (Birt et al., 2021). Due to the unique species and elevated diversity found at this site, a recent (December 2024) extension to the prohibited area for bottom‐towed gears beyond the eastern boundary of the SAC to include the Bristow's to the Stones MCZ is an important step toward management of this site. However, sections of the reef remain completely open to fishing pressure as they fall outside the inshore zone that corresponds to the MCZ boundary (Figure 1C) and similar spatially isolated and topographically complex sites, such as the Pol Bank (Exeter et al., 2024), that are not currently covered by any MPA designation and could be prioritized for further research (Birt et al., 2021; Brown et al., 2022).

In comparison, SAC‐designated sediment habitats held significantly lower levels of richness and biomass but elevated abundance (Table 1). This abundance was attributed to large shoals of *A. tobianus* (lesser sand eel) and aggregations of *C. pagurus* (brown crab) in bare sediments as well as shoals of smaller size class *P. pollachius* within seagrass beds. These areas, which are associated with a mosaic of nearshore habitats such as seagrass, intertidal reefs, and bare sediments, likely represent important foraging sites for the archipelago's nationally important seabird assemblage (Heaney et al., 2008), which has seen considerable declines in recent decades (Heaney & St Pierre, 2017). The presence of large shoals of juvenile fish (unidentified to a species level) coupled with smaller *P. pollachius* also suggests evidence of seagrass beds acting as an important nursery habitat for fish assemblages locally. While the effect of seagrass habitat type was not explicitly modeled in this study (due to limited sample size; *n* = 24), further research into the role of local and regional seagrass beds as nursery habitats, and their connectivity to reef habitats for population recruitment, is warranted (Swadling et al., 2022). These contrasting results between richness and abundance measures underline the benefits of conducting surveys at an ecosystem scale, incorporating a mosaic of habitat to capture diversity and ecosystem processes. Depth was consistently found to perform well as a predictor of richness, abundance, and biomass within the archipelago and was an important driver of body length for several indicator taxa. These results are supported by comparable BRUV studies on the drivers of fish assemblages at both isolated island sites (Brown et al., 2022; Porriños et al., 2024) and within MPAs (Goetze et al., 2021), which found abundance and reserve effectiveness to increase with depth. The effect of depth is likely explained by ontogenetic shifts, processes where larger individuals migrate to deeper water as they grow and age (Goetze et al., 2021) and the cryptic nature of many smaller species in seagrass and kelp forests (i.e., invertebrates) that are not easily detected in BRUV data (Lowry et al., 2012).

Of the other predictor variables, SST performed poorly in modeling ecological indices (Table 2), likely reflecting the smaller fluctuation in SST across a year (range ~8°C) than that of mainland UK. Given the highly offshore positioning of the archipelago, it was surprising that the exposure variable also performed poorly in modeling the ecological indices (Friedlander et al., 2003), aside from negatively influencing body size in both *S. canicula* (small‐spotted catsharks) and *L. bergylta* (ballan wrasse; Figure 6A,E). The lack of exposure effect may be attributed to variation from high to low exposed sites often occurring within short distances of one another throughout the archipelago (Warwick et al., 2003). Remoteness, however, was found to predict elevated levels of biomass and increased body size for both *P. pollachius* and *L. bergylta* (Figure 6D) even when excluding the Seven Stone's deployments from the analysis. This supports recent studies documenting the proximity effect of human activity on the size structure of marine communities (Birt et al., 2021; Murray et al., 2023). Given the limited spatial extent of this study, commercial fishing pressures are present across all deployment locations (Morcom et al., 2023); ecological benefits of remoteness likely relate to reduced recreational fishing and cumulative pressures such as vessel noise pollution (Exeter et al., 2025).

### Application

To date, the majority of stereo BRUV studies and surveys using spatially robust sampling techniques have been conducted in Australasian waters (Letessier et al., 2024; Whitmarsh et al., 2017), with only a handful of published stereo studies in UK waters, which are comprised of smaller sample sizes (i.e., <90; Clark et al., 2024; Hall et al., 2021; Unsworth et al., 2014). BRUV systems (mono and stereo) are not currently prioritized as a monitoring tool within domestic English waters (Noble‐James et al., 2023), despite their proven application for generating holistic monitoring data in both UK (Blampied et al., 2022; Davies et al., 2021) and international contexts (Whitmarsh et al., 2017). The low‐impact, multispecies nature of stereo BRUV systems could support more standardized monitoring of UK marine ecosystems, which currently rely on varied methodologies and disparate datasets (Noble‐James et al., 2023). Their ability to build robust initial datasets with sufficient statistical power to detect relatively subtle changes in ecological metrics also makes them well suited to developing marine monitoring benchmarks in temperate systems (Bicknell et al., 2019). Given UK marine management is expected to shift from its current feature‐based approach to a whole‐site or ecosystem‐based strategy (Davies, Holmes, Attrill, & Sheehan, 2022; Davies, Holmes, Bicknell, et al., 2022; Rees et al., 2020), coupled with the implementation of Highly Protected Marine Areas (Benyon, 2019), stereo BRUV systems are likely to become increasingly important as a complement monitoring tool for MPAs. BRUV systems are generally considered a lower cost option for marine monitoring, so they provide an efficient method for collecting long‐term datasets, as evidenced by a 10‐year monitoring time series in the Lyme Bay SAC (Davies, Holmes, Attrill, & Sheehan, 2022; Davies, Holmes, Bicknell, et al., 2022).

Further application and standardization of BRUV surveys across other UK sites on a scale similar to that which has been adopted for the UK's Overseas Territories (Meeuwig et al., 2021) will also contribute to more effective regional and national assessments of the health of UK marine ecosystems (Goetze et al., 2021) such as assessing progress toward achieving Good Environmental Status (DEFRA, 2019). Lessons learnt from regional, multisite meta‐analyses in turn have the potential to increase the impact of future MPA designations or management interventions at existing sites (Claudet et al., 2010; Pomeroy et al., 2005). The BRUV data presented can also act as an opportunistic sampling method for conservation priority sessile features (i.e., *Eunicella verrucosa*) that have been the focus of dive, ROV, and drop camera efforts previously (Axelsson et al., 2014; Eggleton & Meadows, 2013).

This survey represents one of the most spatially comprehensive uses of BRUV technology in domestic UK waters to date, being among the few that apply stereo BRUV technology and, to our knowledge, the first to employ robust GRTS sampling techniques (Smith et al., 2017). Rarefaction analysis revealed that 51.9 (range: 48.6–55.3) of the 64 species observed would be expected to occur within 100 samples (Figure 7), with 61 (56.6–65.5) species observed within 200 samples. This suggests that the sampling conducted here was more than sufficient, although further efforts in sediment habitats might yield additional species (e.g., *Rajidae* spp.; Herdson, 2010). The presence of spatial autocorrelation in the results, despite using a spatially balanced survey design (GRTS), suggests that wider spacing (>200 m) between deployments would be beneficial in future UK BRUV studies. As the archipelago is a closed system with multiple habitat types within a geographically limited area (Warwick et al., 2003), species accumulation rates may be higher, and sampling more prone to spatial autocorrelation than in less connected mainland UK sites. However, our results support the approach of generating a more modest number of initial GRTS‐generated deployment locations, with spacing between ~500 m, and increasing sampling based on iteratively generated accumulation curves. Stereo BRUV systems were able to measure a modest 14.2% of individuals recorded across all taxa in the 241 stereo deployments. Measurement rates were far higher among commercially targeted species that are more commonly recorded in fisheries‐dependent landings datasets, including such as *P. pollachius* (47.1% of individuals) and *S. canicula* (71.1%). These results support the wider adoption of stereo BRUV systems for providing length and biomass estimates for commercial species where extractive methodologies may not be appropriate or align with the objectives of MPAs (Trenkel et al., 2019).

Locally, the results of this survey can serve as a contemporary benchmark for the Isles of Scilly inshore zone. The versatility of BRUV systems allows the data to be applied at an ecosystem level, supporting a holistic understanding of the health of MPA‐designated reef and sediment habitats (English Nature, 2000), or to inform species‐specific monitoring, such as providing fisheries‐independent data on *P. elephas* populations within MCZs (Natural England, 2019). The comprehensive nature of this survey means that the results can also inform future evidence‐based monitoring strategies and research priorities for these MPAs or other features of interest. BRUV data can also complement more established methods (Honeyman et al., 2023; Jackson‐Bué et al., 2023) by providing data for more inaccessible locations, such as deep, exposed reefs. These methods include SeaSearch (Jackson, 2021) or Natural England underwater dive surveys (Axelsson et al., 2014) which provide information on the health and distribution of sessile and low‐mobility species that are rarely detected by BRUV systems and are also key indicators of ecosystem health. BRUV data can therefore align data levels for marine fish, elasmobranch, and crustacean assemblages with designated features such as seabirds (Heaney & St Pierre, 2017), seals (Sayer & Witt, 2019), seagrass (Bull & Kenyon, 2021), and associated communities of infauna (Warwick & Somerfield, 2015) and corals (Eggleton & Meadows, 2013). More broadly, benchmark values for species richness, abundance, and biomass can be used to measure ecosystem or species‐specific changes in response to impacts such as climate change, marine heatwaves, fisheries practices, or management interventions by replicating this comprehensive survey at appropriate intervals in the future.

## AUTHOR CONTRIBUTIONS

Owen M. Exeter, Kristian Metcalfe, Annette C. Broderick, and Paul J. Somerfield conceived the study. Owen M. Exeter, Francesco Garzon, Tom Hooper, Ricky Pender, and Sarah Morcom conducted the data collection. Owen M. Exeter conducted analyses, synthesized findings, and wrote the first draft with advice from Xavier A. Harrison, Annette C. Broderick, and Kristian Metcalfe, and all authors contributed to the revision of the manuscript and gave approval for publication. Paul J. Somerfield's approval for publication was provided by Plymouth Marine Laboratory.

## CONFLICT OF INTEREST STATEMENT

The authors declare no conflicts of interest.

## ETHICS STATEMENT

All research was subject to ethical approval by the University of Exeter Ethics Committee (Application ID: 492660).

## Supporting information


Appendix S1:

## Data Availability

Data and code (owenex, 2025) are available in Zenodo at https://doi.org/10.5281/zenodo.16367428. Marine habitat data used to inform sampling design was downloaded from the Natural England Marine GI Team internal database by contacting marinegidata@naturalengland.org.uk and requesting “Marine Evidence Base (Internal) dataset 2021.”
