# Supplementary material for: Application of spatially robust stereo‐BRUV sampling for quantifying fish assemblages in UK marine protected areas
Source: Ecol Appl. 2025 Sep 12;35(6):e70104. doi: 10.1002/eap.70104 (PMC12426589; doi:10.1002/eap.70104)
Supplement: Supplementary file 1 — Appendix S1: [file EAP-35-e70104-s001.pdf]

Appendix S1

Application of spatially robust stereo-BRUV sampling for quantifying fish assemblages in UK marine protected areas. Owen M. Exeter, Annette C. Broderick, Xavier A. Harrison, Francesco Garzon, Sarah Morcom, Ricky Pender, Trudy Russell, Ian Saunders, Paul J. Somerfield, Kate Sugar, Colin Trundle, Julie Webber, Tom Hooper, Kristian Metcalfe. *Ecological Applications.*

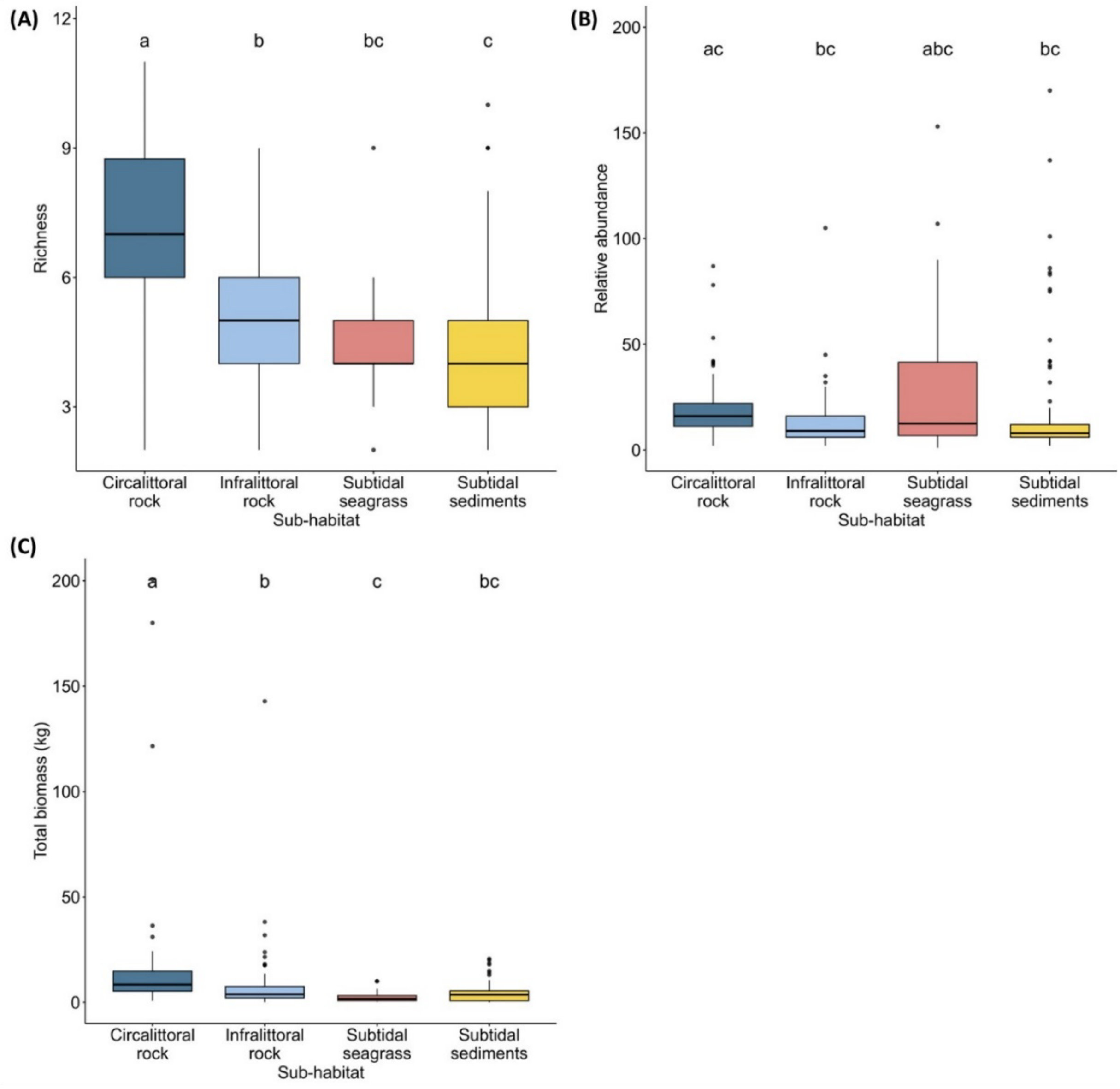

Figure S1. Box and whiskers plot showing sub-feature habitat (A) demersal species richness; (B) demersal relative abundance and (C) total biomass. Letters indicate the results of the Kruskal-Wallis and Dunn's pairwise post-hoc tests (Bonferroni adjustment).

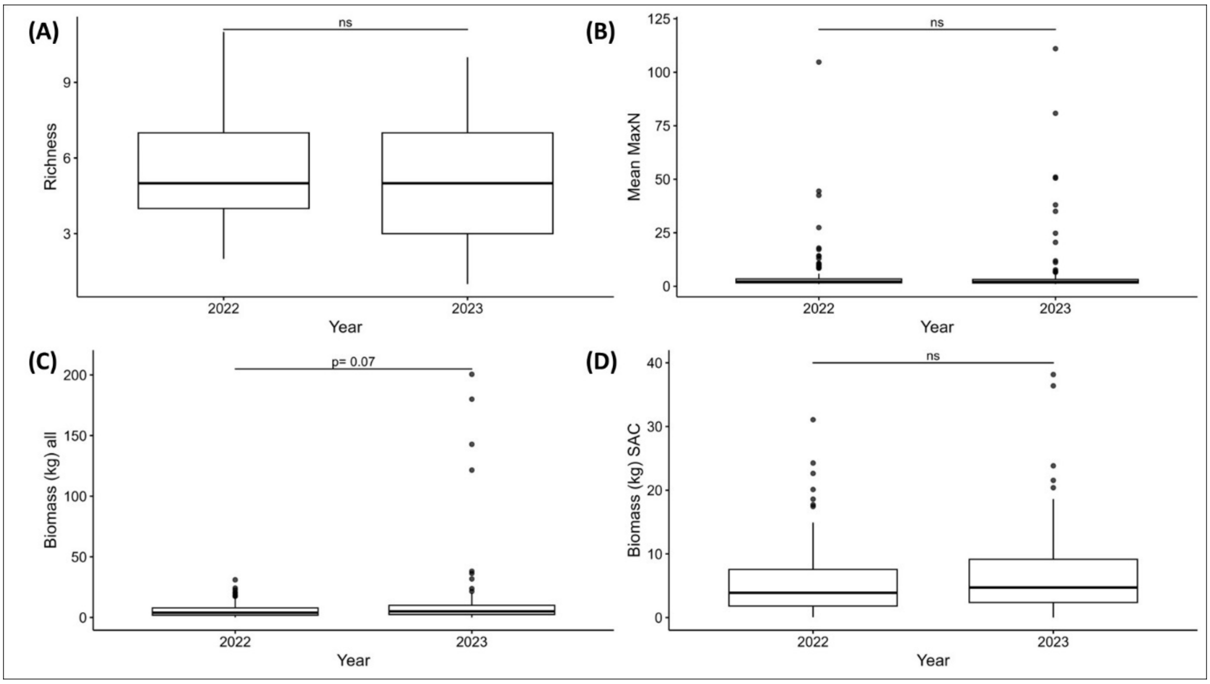

Figure S2. Box and whiskers plot showing comparison of year effect on (A) demersal species richness; (B) demersal relative abundance; (C) total biomass and (D) total biomass within the SAC only. Results of the Mann-Whitney U test shown.

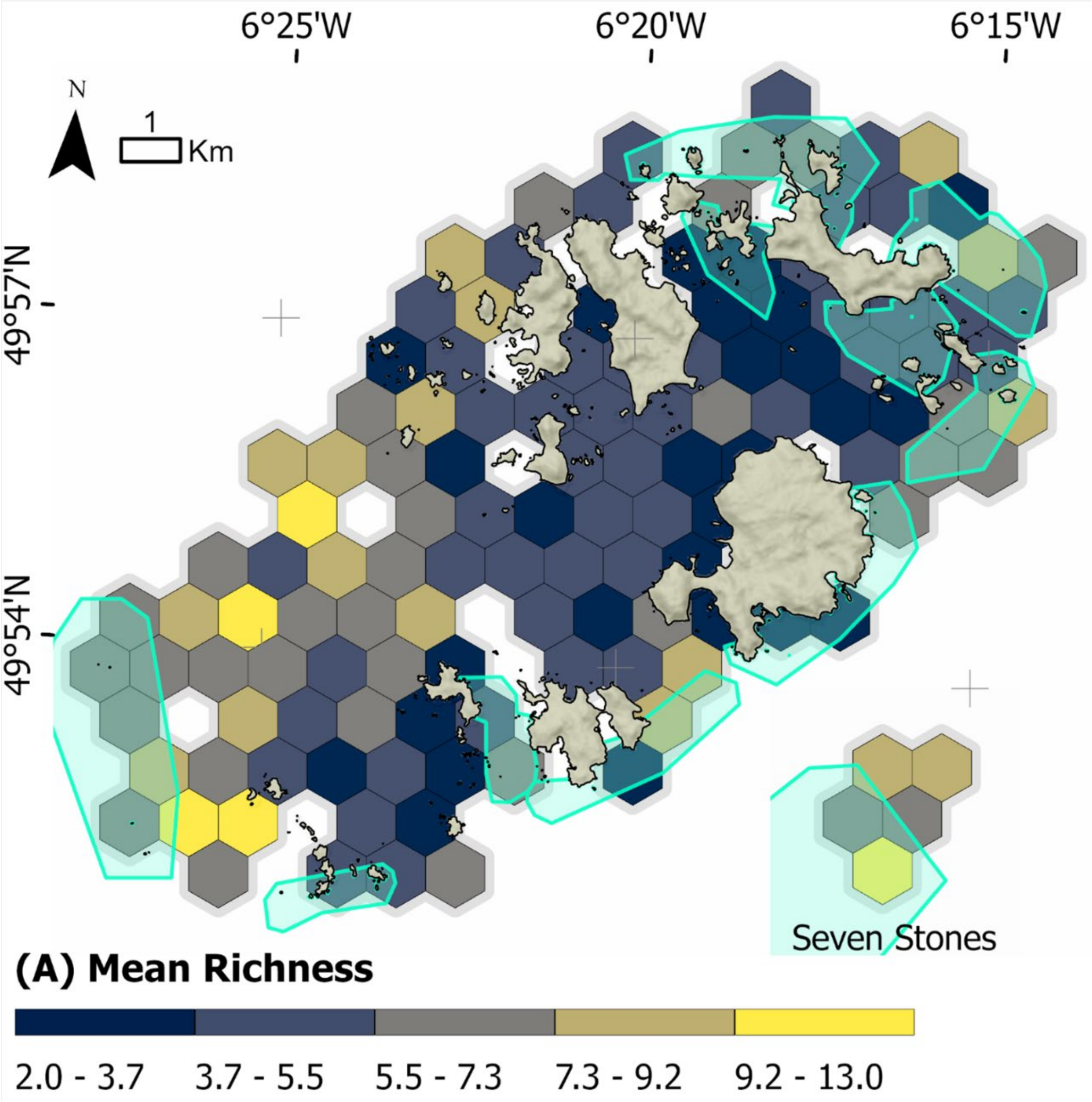

Figure S3. Mean values for demersal richness deployments at a 1 km2 hexagonal grid cell resolution as per Figure 4, with Marine Conservation Zone sites overlaid (green polygons).

Table S1. Predictor variables used in the model selection for species richness, relative abundance and biomass metrics, outlining where the data source.

Variable	Unit	Method	Resolution	Mean	Min	Max
**Retained**						
Depth	Meters	In situ	Site	21.4	1.6	44.0
Temperature	Degree Celsius	In situ	Site	14.4	11.0	17.3
Relief score	0 - 5	In situ	Site	1.9	0.0	5.0
Remoteness	Meters	GIS	Site	2790.4	13420.0	32.1
Exposure	Degrees	GIS	200 m	89.4	0.7	175.6
**Excluded**						
Slope mean	Degrees	GIS	200 m	4.1	0.1	18.7
Ruggedness range	Meters	GIS	200 m	6.8	0.2	27.1

Table S2. Pearson's correlation results for all predictor variables. Discarded predictors highlighted in bold.

Variable	Relief score	Depth	Temp	Rug range	Remoteness	Exposure	Slope mean
Relief score	1.00	0.52	0.00	0.57	0.45	0.11	0.64
Depth	0.52	1.00	0.00	0.36	0.50	0.08	0.52
Temp	0.00	0.00	1.00	-0.04	0.19	0.03	0.16
**Rug range**	**0.57**	**0.36**	**-0.04**	**1.00**	**0.05**	**-0.16**	**0.56**
Remoteness	0.45	0.50	0.19	0.05	1.00	0.17	0.68
Exposure	0.11	0.08	0.03	-0.16	0.17	1.00	0.02
**Slope mean**	**0.64**	**0.52**	**0.16**	**0.56**	**0.68**	**0.02**	**1.00**

Table S3. Variable inflation factor (VIF) between all predictors of demersal species richness, demersal abundance and total biomass. Discarded predictors (VIF exceeded five) highlighted in bold.

VARIABLES	Richness	MaxN	Diversity	Biomass
Relief score	2.42	2.42	2.42	2.42
Depth	1.65	1.65	1.65	1.65
Temp	1.09	1.09	1.09	1.09
**Ruggedness range**	**2.77**	**2.77**	**2.77**	**2.77**
Remoteness	3.28	3.28	3.28	3.28
Exposure	1.13	1.13	1.13	1.13
**Slope mean**	**4.15**	**4.15**	**4.15**	**4.15**

Table S4. Generalised linear models (GLMs) variables and interactions results, with the highest ranked models within two units of AICc for predicting the demersal species richness, relative abundance and total species biomass highlighted in bold. Included model terms indicated by + symbol.

Response	Intercept	CI lower	CI upper	Predictor*											df	logLik	AICc	Delta	Weight	Pseudo R^2
				Habitat	Depth	Temp	Relief score	Remoteness	Exposure	Depth: Habitat	Habitat: Temp	Habitat: Relief score	Habitat: Remoteness	Exposure: Habitat						
**Richness**	**1.09**	**0.88**	**1.30**		**+**		**+**								**9**	**-565.5**	**1149.6**	**0.00**	**0.48**	**0.390**
**Richness**	**1.18**	**0.95**	**1.42**	**+**	**+**					**+**					**6**	**-568.8**	**1149.9**	**0.30**	**0.42**	**0.365**
Richness	1.15	0.94	1.36		+			+							5	-571.9	1154.1	4.49	0.05	0.341
Richness	1.08	0.89	1.28		+										4	-573.2	1154.5	4.97	0.04	0.331
Richness	1.08	0.44	1.73	+		+					+				6	-573.0	1158.4	8.80	0.01	0.333
Richness	1.13	0.91	1.35				+	+							9	-571.0	1160.6	11.00	0.00	0.348
Richness	1.06	0.85	1.27				+								8	-572.2	1161.0	11.44	0.00	0.339
Richness	1.09	0.86	1.32				+		+						9	-572.1	1162.8	13.22	0.00	0.340
Richness	1.20	0.99	1.42	+											4	-578.3	1164.8	15.26	0.00	0.292
Richness	1.28	1.04	1.51	+				+					+		6	-576.3	1164.9	15.37	0.00	0.308
Richness	0.95	0.34	1.55	+			+					+			10	-572.2	1165.1	15.56	0.00	0.339
Richness	1.21	0.98	1.45	+					+					+	6	-577.8	1167.9	18.38	0.00	0.296
Richness	1.12	0.91	1.34					+							4	-581.6	1171.4	21.81	0.00	0.267
Richness	0.45	-0.09	1.00			+									4	-582.2	1172.5	22.97	0.00	0.263
Richness	1.16	0.93	1.39					+	+						5	-581.3	1172.9	23.31	0.00	0.270
Richness	1.03	0.82	1.24						+						4	-584.5	1177.1	27.57	0.00	0.245
Richness	1.69	1.64	1.74												1	-616.8	1235.6	86.02	0.00	0.000
**Total maxN**	**2.07**	**1.73**	**2.41**	**+**	**+**					**+**					**7**	**-1113.1**	**2240.5**	**0.00**	**0.98**	**0.339**
Total maxN	2.80	2.53	3.07		+		+								10	-1113.7	2248.3	7.77	0.02	0.336
Total maxN	2.46	2.23	2.68	+				+					+		7	-1131.9	2278.2	37.72	0.00	0.250
Total maxN	2.75	2.38	3.11	+					+					+	7	-1134.2	2282.9	42.38	0.00	0.238
Total maxN	2.62	2.43	2.82	+											5	-1136.9	2283.9	43.41	0.00	0.225
Total maxN	2.21	0.77	3.65	+		+					+				7	-1135.3	2285.0	44.47	0.00	0.233
Total maxN	3.36	3.10	3.62				+	+							10	-1135.0	2290.9	50.34	0.00	0.234
Total maxN	1.55	0.42	2.69	+			+					+			11	-1134.0	2290.9	50.40	0.00	0.240
Total maxN	3.35	3.09	3.62				+								9	-1139.8	2298.3	57.81	0.00	0.210
Total maxN	3.26	2.91	3.60				+		+						10	-1139.5	2299.8	59.32	0.00	0.211
Total maxN	2.48	2.22	2.73		+			+							6	-1145.2	2302.8	62.22	0.00	0.181
Total maxN	2.50	2.24	2.75		+										5	-1146.6	2303.5	62.95	0.00	0.174
Total maxN	2.38	1.17	3.59			+									5	-1154.7	2319.7	79.13	0.00	0.129

Total maxN	2.89	2.68	3.09					+							5	-1155.0	2320.2	79.69	0.00	0.128
Total maxN	2.87	2.56	3.18						+						5	-1155.0	2320.2	79.69	0.00	0.128
Total maxN	2.87	2.55	3.18					+	+						6	-1155.0	2322.3	81.75	0.00	0.128
Total maxN	3.18	3.06	3.31												2	-1176.3	2356.6	116.05	0.00	0.000
**Total biomass**	**0.58**	**0.32**	**0.85**		**+**										**5**	**-703.8**	**1417.9**	**0.00**	**0.40**	**0.229**
**Total biomass**	**0.61**	**0.35**	**0.88**		**+**			**+**							**6**	**-703.1**	**1418.5**	**0.64**	**0.29**	**0.233**
**Total biomass**	**0.66**	**0.27**	**1.04**	**+**	**+**					**+**					**7**	**-702.3**	**1419.0**	**1.12**	**0.23**	**0.237**
Total biomass	0.44	0.14	0.73		+		+								10	-700.1	1421.0	3.14	0.08	0.248
Total biomass	1.22	0.94	1.50	+				+					+		7	-714.2	1442.9	25.05	0.00	0.174
Total biomass	1.21	0.94	1.48	+											5	-716.9	1444.0	26.18	0.00	0.160
Total biomass	1.74	0.17	3.30	+		+					+				7	-715.7	1445.8	27.89	0.00	0.167
Total biomass	1.20	0.86	1.55					+	+						6	-716.9	1446.1	28.26	0.00	0.160
Total biomass	1.41	0.99	1.83	+					+					+	7	-715.9	1446.2	28.32	0.00	0.165
Total biomass	1.01	0.78	1.25					+							5	-718.2	1446.7	28.87	0.00	0.152
Total biomass	0.85	0.58	1.12				+	+							10	-713.6	1448.0	30.12	0.00	0.178
Total biomass	0.84	0.57	1.10				+								9	-715.1	1449.0	31.14	0.00	0.169
Total biomass	1.69	0.61	2.77	+			+					+			11	-713.2	1449.4	31.58	0.00	0.180
Total biomass	0.95	0.58	1.32				+		+						10	-714.7	1450.3	32.44	0.00	0.172
Total biomass	1.16	0.82	1.50						+						5	-720.5	1451.3	33.45	0.00	0.140
Total biomass	0.86	-0.37	2.08			+									5	-721.5	1453.2	35.34	0.00	0.134
Total biomass	1.80	1.68	1.92												2	-743.8	1491.7	73.80	0.00	0.000

*all models include the spatially lagged term and year variables

Table S5. Results of Moran's Index test results for generalised linear mixed models with site coordinates (Lat, Lon) included as a random effect using the ‘fitme’ function from the spaMM R package. Bold values indicate significance (<0.05).

Terms*	Richness			Total MaxN			Total biomass (kg)		
	Moran's Index	Sd	p-value	Moran's Index	Sd	p-value	Moran's Index	Sd	p-value
null_model	**0.355**	**9.08**	**0.00**	**0.187**	**4.91**	**0.00**	**0.187**	**4.91**	**0.00**
Habitat	**0.076**	**2.02**	**0.02**	**0.247**	**6.37**	**0.00**	-0.005	-0.04	0.52
Depth	0.047	1.29	0.10	**0.077**	**2.03**	**0.02**	**0.101**	**2.66**	**0.00**
Temp	**0.067**	**1.79**	**0.04**	**0.149**	**3.86**	**0.00**	-0.058	-1.38	0.92
Relief score	**0.092**	**2.41**	**0.01**	0.022	0.65	0.26	-0.013	-0.23	0.59
Remoteness	**0.081**	**2.14**	**0.02**	**0.094**	**2.47**	**0.01**	-0.032	-0.72	0.76
Exposure	**0.067**	**1.79**	**0.04**	**0.122**	**3.23**	**0.00**	-0.046	-1.08	0.86
Depth * Habitat	**0.073**	**1.94**	**0.03**	0.055	1.48	0.07	**0.090**	**2.36**	**0.01**
Temp * Habitat	**0.064**	**1.72**	**0.04**	0.018	0.56	0.29	-0.010	-0.16	0.56
Relief score * Habitat	**0.092**	**2.42**	**0.01**	-0.018	-0.37	0.64	-0.013	-0.23	0.59
Remoteness * Habitat	**0.091**	**2.40**	**0.01**	-0.001	0.07	0.47	-0.033	-0.76	0.78
Exposure * Habitat	**0.068**	**1.83**	**0.03**	0.054	1.46	0.07	-0.007	-0.10	0.54
Relief score + Depth	**0.108**	**2.83**	**0.00**	0.037	1.04	0.15	**0.071**	**1.90**	**0.03**
Relief score + Remoteness	**0.135**	**3.51**	**0.00**	-0.010	-0.16	0.57	-0.043	-1.00	0.84
Relief score + Exposure	**0.092**	**2.42**	**0.01**	**0.174**	**4.53**	**0.00**	-0.055	-1.30	0.90
Remoteness + Exposure	**0.079**	**2.08**	**0.02**	**0.118**	**3.13**	**0.00**	-0.029	-0.64	0.74
Depth + Remoteness	**0.070**	**1.85**	**0.03**	**0.145**	**3.83**	**0.00**	**0.091**	**2.40**	**0.01**

* All models control for the effect of year and include the 'Matern(1|Lat + Lon)' term as a random factor

Table S6. Summary of BRUVS deployments by feature and sub-feature habitat types.

Habitat	Deployments					MPA		In situ variables measured			Data collection		
	Total	2022	2023	Stereo	Mono	SAC (n.)	MCZ (n.)	Mean depth (m)	Mean Temp (deg)	Mean relief	Start	End	Days (n.)
Reef	181	93	88	137	44	167	50	22.4	14.3	2.5	20/05/2022	28/08/2023	47
Circalittoral rock	82	40	42	66	16	72	27	29.3	14.3	3.1	24/05/2022	28/08/2023	26
Infralittoral rock	99	53	46	71	28	95	23	16.7	14.4	2.0	20/05/2022	28/08/2023	42
Subtidal sediments	99	42	57	83	16	98	27	14.8	14.0	0.2	19/05/2022	28/08/2023	37
Seagrass	24	18	6	22	2	24	11	5.3	14.8	0.1	13/06/2022	04/07/2023	15
Sediments (other)	75	24	51	61	14	74	16	17.8	13.8	0.2	19/05/2022	28/08/2023	29

Note: mono deployments include 21 stereo BRUVS where obstruction prevented any observations or measurements from one of the cameras

Table S7. Summary of observed species including mean relative abundance across all 280 BRUVS deployments.

Family	Species	Species common name	Simplified functional group	Mean MaxN	SD	Total MaxN
Ammodytidae	*Ammodytes tobianus*	Lesser sandeel	Demersal fish	11.70	47.97	3277
	Hyperoplus lanceolatus	Greater sandeel	Benthopelagic fish	0.03	0.25	7
Asteriidae	*Asterias rubens*	Common starfish	Demersal invertebrates	0.03	0.20	9
	Marthasterias glacialis	Spiny starfish	Demersal invertebrates	0.17	0.49	47
Atherinidae	*Atherina presbyter*	Sand smelt	Pelagic fish	0.02	0.25	5
Blenniidae	*Parablennius gattorugine*	Tompot blenny	Demersal fish	0.01	0.10	3
Callionymidae	*Callionymus lyra*	Common dragonet	Demersal fish	0.13	0.60	37
Cancridae	*Cancer pagurus*	Edible crab	Crustaceans	0.46	1.02	128
Caproidae	*Capros aper*	Boarfish	Demersal fish	0.09	1.43	24
Carangidae	*Trachurus trachurus*	Atlantic horse mackerel	Pelagic fish	4.57	53.79	1280
Carcinidae	*Carcinus maenas*	Shore crab	Crustaceans	0.54	1.60	152
Clupeidae	*Sardina pilchardus*	European pilchard	Pelagic fish	0.07	0.97	19
Congridae	*Conger conger*	Conger eel	Demersal fish	0.12	0.33	33
Corystidae	*Corystes cassivelaunus*	Masked crab	Crustaceans	0.01	0.08	2
Delphinidae	*Delphinus delphis*	Common dolphin	Cetacean	0.00	0.06	1
Echinidae	*Echinus esculentus*	Common urchin	Demersal invertebrates	0.34	0.91	96
Gadidae	*Gadus morhua*	Atlantic cod	Benthopelagic fish	0.01	0.15	4
	Merlangius merlangus	Whiting	Benthopelagic fish	0.01	0.12	2
	Pollachius pollachius	Pollack	Benthopelagic fish	3.27	5.09	916
	Pollachius virens	Saithe	Demersal fish	0.04	0.22	10
	Trisopterus luscus	Poor cod	Demersal fish	0.53	1.62	147
	Trisopterus minutus	Pouting	Benthopelagic fish	0.14	0.85	40
Gaidropsaridae	*Gaidropsarus vulgaris*	Three bearded rockling	Demersal fish	0.06	0.26	18
Gasterosteidae	*Spinachia spinachia*	Fifteen spined stickleback	Reef associated fish	0.03	0.23	9
Gobiidae	*Gobiusculus flavescens*	Two spotted goby	Reef associated fish	0.03	0.28	8
	Pomatoschistus minutus	Sand goby	Demersal fish	0.30	2.23	83
	Thorogobius ephippiatus	Leopard spotted goby	Demersal fish	0.01	0.08	2
Holothuriidae	*Holothuria forskali*	Cotton spinner	Demersal invertebrates	0.05	0.28	13
Labridae	*Acantholabrus palloni*	Scale rayed wrasse	Reef associated fish	0.00	0.06	1
	Centrolabrus exoletus	Rock cook	Reef associated fish	0.89	2.34	250
	Ctenolabrus rupestris	Goldsinny wrasse	Reef associated fish	0.78	1.24	218
	Labrus bergylta	Ballan wrasse	Reef associated fish	1.28	1.72	359
	Labrus mixtus	Cuckoo wrasse	Reef associated fish	1.05	1.46	294

	Symphodus melops	Corkwing wrasse	Reef associated fish	0.38	0.98	106
Loliginidae	*Loligo vulgaris*	Common squid	Cephalopods	0.00	0.06	1
Lotidae	*Molva molva*	Ling	Demersal fish	0.01	0.10	3
Majidae	*Maja brachydactyla*	Spiny spider crab	Crustaceans	0.19	0.47	54
Mugilidae	*Chelon labrosus*	Thicklip mullet	Demersal fish	0.03	0.25	8
Mullidae	*Mullus surmuletus*	Red mullet	Demersal fish	0.17	1.30	48
Muricidae	*Nucella lapillus*	Dog whelk	Demersal invertebrates	0.50	2.96	141
Nephropidae	*Homarus gammarus*	European lobster	Crustaceans	0.09	0.30	24
Octopodidae	*Octopus vulgaris*	Common octopus	Cephalopods	0.00	0.06	1
Paguridae	*Pagurus bernhardus*	Hermet crab	Crustaceans	0.02	0.15	6
Palaemonidae	*Palaemon serratus*	Common prawn	Shrimps	0.01	0.13	3
Palinuridae	*Palinurus elephas*	European spiny lobster	Crustaceans	0.20	0.57	57
Phalacrocoracidae	*Phalacrocorax aristotelis*	European shag	Seabirds	0.01	0.08	2
Phocidae	*Halichoerus grypus*	Grey seal	Marine mammals	0.03	0.19	8
Pholidae	*Pholis gunnellus*	Butterfish	Demersal fish	0.00	0.06	1
Polybiidae	*Liocarcinus depurator*	Harbour crab	Crustaceans	0.02	0.16	5
Portunidae	*Necora puber*	Velvet swimming crab	Crustaceans	0.08	0.33	22
Scombridae	*Scomber scombrus*	Atlantic mackerel	Pelagic fish	3.95	59.90	1106
	Thunnus thynnus	Atlantic bluefin tuna	Pelagic fish	0.01	0.08	2
Scyliorhinidae	*Scyliorhinus canicula*	Small spotted catshark	Sharks and rays	0.97	1.22	271
	Scyliorhinus stellaris	Nursehound	Sharks and rays	0.25	0.61	69
Soleidae	*Solea solea*	Common sole	Flatfishes	0.03	0.19	8
Sparidae	*Sparus aurata*	Gilthead bream	Demersal fish	0.04	0.54	10
	Spondyliosoma cantharus	Black seabream	Demersal fish	0.02	0.16	5
Sulidae	*Morus bassanus*	Northern gannet	Seabirds	0.00	0.06	1
Triakidae	*Galeorhinus galeus*	Tope	Sharks and rays	0.00	0.06	1
Triglidae	*Chelidonichthys cuculus*	Red gurnard	Demersal fish	0.00	0.06	1
	Chelidonichthys lastoviza	Streaked gurnard	Demersal fish	0.01	0.08	2
	Chelidonichthys lucerna	Tub gurnard	Demersal fish	0.00	0.06	1
	Eutrigla gurnardus	Grey gurnard	Demersal fish	0.03	0.23	9
Zeidae	*Zeus faber*	John dory	Benthopelagic fish	0.01	0.10	3

Table S8. Length measurements source by true measurement ('Measured') or proxy mean length source for each deployment.

Species	Measured	Proxy habitat	Proxy study	Proxy FishBase	Proxy study family
Acantholabrus palloni	1	0	0	0	0
Ammodytes tobianus	0	2	0	0	0
Ammodytidae	0	0	3	0	0
Atherina presbyter	1	0	1	0	0
Callionymus lyra	0	0	0	19	0
Cancer pagurus	44	32	0	0	0
Capros aper	1	0	0	0	0
Carcinus maenas	23	23	0	0	0
Centrolabrus exoletus	44	41	0	0	0
Chelidonichthys cuculus	1	0	0	0	0
Chelidonichthys lastoviza	1	0	1	0	0
Chelidonichthys lucerna	1	0	0	0	0
Chelon labrosus	2	2	0	0	0
Conger conger	11	21	0	0	0
Ctenolabrus rupestris	44	76	1	0	0
Eutrigla gurnardus	6	1	0	0	0
Gadus morhua	3	0	0	0	0
Gaidropsarus vulgaris	3	10	4	0	0
Galeorhinus galeus	1	0	0	0	0
Gobiusculus flavescens	0	0	0	8	0
Halichoerus grypus	2	2	10	0	0
Homarus gammarus	10	11	1	0	0
Hyperoplus lanceolatus	1	1	0	0	0
Labrus bergylta	116	82	0	0	2
Labrus mixtus	94	49	0	0	0
Maja brachydactyla	18	28	1	0	0
Merlangius merlangus	1	0	0	0	0
Molva molva	2	1	0	0	0
Mullus surmuletus	8	6	0	0	0
Necora puber	4	2	12	0	0
Palinurus elephas	29	13	0	0	0
Parablennius gattorugine	0	0	0	3	0
Pholis gunnellus	0	0	0	1	0

Pollachius pollachius	131	111	0	0	0
Pollachius virens	3	5	0	0	0
Pomatoschistus minutus	0	0	0	14	0
Sardina pilchardus	2	0	0	0	0
Scomber scombrus	6	6	0	0	0
Scyliorhinus canicula	111	39	0	0	0
Scyliorhinus stellaris	25	27	0	0	0
Solea solea	2	5	0	0	0
Sparus aurata	2	0	0	0	0
Spinachia spinachia	1	2	4	0	0
Spondyliosoma cantharus	3	1	0	0	0
Symphodus melops	34	21	1	0	0
Thorogobius ephippiatus	0	0	0	2	0
Thunnus thynnus	1	1	0	0	0
Trachurus trachurus	4	1	0	0	0
Trisopterus luscus	35	15	0	0	0
Trisopterus minutus	14	2	0	0	0
Zeus faber	3	0	0	0	0
Cod spp	0	0	0	0	6
Goby spp	0	0	0	77	0
Sandeel spp	0	0	0	0	54
Wrasse spp	0	0	0	0	91
**Total**	**849**	**639**	**39**	**124**	**153**
**Frequency**	**0.51**	**0.39**	**0.02**	**0.08**	**0.09**

Table S9. Results of Moran's Index test results for models with spatially lagged variable. Bold values indicate significance (<0.05).

Terms	Richness			Total MaxN			Total biomass (kg)		
	Moran's Index	Sd	p-value	Moran's Index	Sd	p-value	Moran's Index	Sd	p-value
null_model	**0.355**	**9.08**	**0.00**	**0.187**	**4.91**	**0.00**	**0.360**	**8.97**	**0.00**
Habitat	-0.106	-2.59	1.00	**0.068**	**1.81**	**0.03**	0.031	0.86	0.19
Depth	-0.045	-1.05	0.85	-0.073	-1.77	0.96	0.011	0.36	0.36
Temp	-0.112	-2.74	1.00	-0.062	-1.49	0.93	0.033	0.90	0.18
Relief score	-0.077	-1.85	0.97	0.044	1.21	0.11	0.040	1.09	0.14
Remoteness	-0.080	-1.94	0.97	-0.060	-1.44	0.93	**0.065**	**1.70**	**0.04**
Exposure	-0.128	-3.16	1.00	-0.057	-1.37	0.91	0.027	0.76	0.22
Depth * Habitat	-0.019	-0.39	0.65	0.011	0.36	0.36	0.021	0.61	0.27
Temp * Habitat	-0.097	-2.35	0.99	0.061	1.65	0.05	0.026	0.72	0.24
Relief score * Habitat	-0.077	-1.85	0.97	**0.065**	**1.75**	**0.04**	0.032	0.87	0.19
Remoteness * Habitat	-0.068	-1.64	0.95	0.031	0.89	0.19	0.049	1.31	0.10
Exposure * Habitat	-0.108	-2.65	1.00	**0.091**	**2.41**	**0.01**	0.028	0.77	0.22
Relief score + Depth	-0.004	0.00	0.50	-0.012	-0.20	0.58	0.025	0.71	0.24
Relief score + Remoteness	-0.049	-1.16	0.88	0.023	0.68	0.25	0.065	1.68	0.05
Relief score + Exposure	-0.078	-1.88	0.97	0.044	1.22	0.11	0.037	1.01	0.16
Remoteness + Exposure	-0.084	-2.04	0.98	-0.059	-1.43	0.92	0.061	1.59	0.06
Depth + Remoteness	-0.023	-0.49	0.69	-0.052	-1.24	0.89	0.026	0.74	0.23

Moran's Index test results for models with spatially laged variable. Bold p=-values indicate significance (>=0.05)

Table S10. Generalised linear models (GLMs) results for indicator species length models, with the highest ranked models within two units of AICc for predicting the body length highlighted in bold. Included model terms indicated by + symbol.

Indicator	(Intercept)	CI lower	CI upper	Habitat	Depth	Temp	Relief score	Remoteness	Exposure	Depth: Habitat	Habitat: Temp	Habitat :Relief_score	Habitat: Remoteness	Exposure: Habitat	df	logLik	AICc	delta	weight	Pseudo r squared
**(A) Small spotted catshark**																				
	**654.96**	**615.83**	**694.09**						**+**						**4**	**-623.1**	**1254.5**	**0.00**	**0.41**	**0.099**
	**653.06**	**613.74**	**692.38**					**+**	**+**						**5**	**-622.6**	**1255.8**	**1.21**	**0.22**	**0.106**
	**672.81**	**623.77**	**721.85**	**+**					**+**					**+**	**6**	**-621.5**	**1255.9**	**1.36**	**0.21**	**0.123**
	645.64	605.20	686.08				+		+						9	-619.0	1257.8	3.26	0.08	0.162
	626.53	590.28	662.77		+	+									4	-626.4	1261.1	6.57	0.02	0.044
	622.01	609.65	634.36												2	-628.9	1261.9	7.37	0.01	0.000
	610.87	586.63	635.10	+											4	-626.8	1262.0	7.49	0.01	0.036
	627.25	590.96	663.53		+			+							5	-625.9	1262.3	7.80	0.01	0.052
	550.11	399.50	700.71			+									4	-627.1	1262.6	8.09	0.01	0.031
	644.07	586.86	701.27	+	+					+					6	-625.0	1262.9	8.31	0.01	0.067
	604.73	578.92	630.54					+							4	-627.4	1263.2	8.61	0.01	0.027
	622.76	585.02	660.49		+		+								9	-622.0	1263.7	9.19	0.00	0.116
	598.66	571.12	626.21				+								8	-623.7	1264.8	10.29	0.00	0.088
	611.07	580.25	641.89	+				+					+		6	-626.4	1265.7	11.13	0.00	0.043
	580.66	395.24	766.07	+		+					+				6	-626.5	1265.8	11.23	0.00	0.042
	601.43	572.99	629.86				+	+							9	-623.4	1266.5	11.99	0.00	0.094
	581.11	488.90	673.32	+			+					+			10	-623.2	1268.5	13.94	0.00	0.097
**(B) Nursehound**																				
	918.63	835.21	1002.04												2	-183.6	371.6	0.00	0.52	0.000
	906.02	732.66	1079.39					+							4	-182.5	374.9	3.23	0.10	0.074
	861.48	707.43	1015.53	+											4	-182.6	375.1	3.44	0.09	0.067
	1237.49	211.24	2263.75			+									4	-182.9	375.6	3.93	0.07	0.050
	911.32	643.78	1178.85		+										4	-183.1	375.9	4.29	0.06	0.037
	861.40	570.84	1151.95						+						4	-183.2	376.2	4.52	0.05	0.029
	924.58	612.60	1236.56					+	+						5	-182.5	377.9	6.25	0.02	0.075
	894.78	624.78	1164.79		+			+							5	-182.5	377.9	6.26	0.02	0.075
	433.50	-148.98	1015.99	+	+					+					6	-181.1	378.5	6.86	0.02	0.164
	1023.86	752.98	1294.75				+								7	-180.2	380.3	8.64	0.01	0.221
	891.78	707.46	1076.11	+				+					+		6	-182.2	380.7	9.05	0.01	0.094
	806.09	474.02	1138.17	+					+					+	6	-182.3	380.9	9.25	0.01	0.087
	1055.68	-362.15	2473.52	+		+					+				6	-182.5	381.1	9.49	0.00	0.079

	1033.82	763.93	1303.70				+	+							8	-179.4	382.8	11.12	0.00	0.267
	961.73	399.59	1523.87	+			+					+			8	-180.2	384.3	12.66	0.00	0.224
	1041.29	718.62	1363.95		+		+								8	-180.2	384.3	12.69	0.00	0.223
	1040.64	660.48	1420.81				+		+						8	-180.2	384.4	12.73	0.00	0.222
**(C) Pollock**																				
	**80.77**	**47.84**	**113.71**		**+**			**+**							**5**	**-776.0**	**1562.5**	**0.00**	**1.00**	**0.700**
	77.97	42.11	113.84		+										4	-787.8	1583.9	21.33	0.00	0.641
	71.02	25.47	116.57		+		+								9	-783.4	1586.3	23.72	0.00	0.664
	84.23	36.24	132.22	+	+					+					6	-787.2	1587.1	24.55	0.00	0.644
	144.92	97.89	191.95				+	+							9	-799.6	1618.7	56.20	0.00	0.570
	212.85	176.31	249.39	+				+					+		6	-807.1	1626.9	64.39	0.00	0.518
	187.97	155.86	220.07					+							4	-811.6	1631.6	69.02	0.00	0.483
	169.55	113.42	225.69					+	+						5	-811.3	1633.1	70.55	0.00	0.486
	174.30	-60.78	409.39	+			+					+			10	-808.3	1638.5	75.93	0.00	0.509
	120.17	52.31	188.02				+		+						9	-814.2	1648.0	85.43	0.00	0.462
	156.96	104.20	209.72				+								8	-815.7	1648.6	86.06	0.00	0.450
	205.31	127.39	283.23	+					+					+	6	-832.0	1676.7	114.17	0.00	0.295
	630.50	290.17	970.83	+		+					+				6	-832.3	1677.2	114.68	0.00	0.292
	268.92	230.18	307.65	+											4	-834.6	1677.6	115.07	0.00	0.266
	166.39	99.15	233.63						+						4	-835.5	1679.3	116.74	0.00	0.256
	510.03	202.82	817.23			+									4	-837.4	1683.2	120.66	0.00	0.234
	320.76	292.37	349.15												2	-854.9	1713.8	151.30	0.00	0.000
**(D) Ballan wrasse**																				
	**318.34**	**271.91**	**364.77**					**+**	**+**						**5**	**-697.9**	**1406.4**	**0.00**	**0.35**	**0.098**
	**317.89**	**271.00**	**364.78**						**+**						**4**	**-699.6**	**1407.6**	**1.16**	**0.20**	**0.072**
	277.75	248.09	307.41					+							4	-700.4	1409.1	2.73	0.09	0.059
	163.33	-52.64	379.30			+									4	-700.5	1409.3	2.93	0.08	0.058
	289.91	253.43	326.40		+			+							5	-699.7	1410.0	3.63	0.06	0.070
	288.79	252.09	325.49		+										4	-701.0	1410.5	4.05	0.05	0.049
	281.70	250.09	313.32	+											4	-701.1	1410.5	4.06	0.05	0.049
	331.02	274.22	387.82	+					+					+	6	-698.9	1410.5	4.07	0.05	0.084
	264.99	227.99	301.98	+				+					+		6	-699.6	1411.9	5.50	0.02	0.073
	310.37	292.30	328.44												2	-704.0	1412.1	5.65	0.02	0.000
	202.95	-54.34	460.23	+		+					+				6	-699.9	1412.5	6.14	0.02	0.067
	270.93	219.12	322.73	+	+					+					6	-700.4	1413.5	7.11	0.01	0.060
	311.31	253.56	369.07				+		+						9	-698.0	1415.7	9.28	0.00	0.097

		283.56	233.50	333.62				+								8	-699.8	1416.9	10.48	0.00	0.069
		281.65	231.56	331.74				+	+							9	-699.1	1417.8	11.38	0.00	0.081
		288.58	237.17	340.00		+		+								9	-699.4	1418.4	12.04	0.00	0.076
		269.22	177.94	360.49	+			+					+			9	-699.7	1419.1	12.68	0.00	0.070
**(E)**	**Cuckoo wrasse**																				
		**267.78**	**211.35**	**324.22**	**+**	**+**					**+**					**6**	**-494.0**	**1001.0**	**0.00**	**0.99**	**0.257**
		237.79	218.16	257.43	+											4	-502.4	1013.2	12.19	0.00	0.113
		208.75	73.00	344.51	+		+					+				6	-500.4	1013.8	12.75	0.00	0.149
		142.37	77.67	207.06		+		+								9	-496.9	1014.0	13.02	0.00	0.209
		241.13	218.50	263.75	+				+					+		6	-500.8	1014.5	13.53	0.00	0.142
		178.55	127.69	229.41				+								8	-498.6	1014.8	13.82	0.00	0.182
		195.54	148.05	243.04		+										4	-503.4	1015.3	14.26	0.00	0.093
		126.95	33.39	220.51	+			+					+			9	-497.7	1015.5	14.48	0.00	0.197
		188.26	133.10	243.41				+		+						9	-498.1	1016.4	15.40	0.00	0.189
		252.38	218.43	286.32	+					+					+	6	-501.8	1016.6	15.58	0.00	0.123
		179.32	128.16	230.48				+	+							9	-498.4	1017.0	15.98	0.00	0.184
		195.83	148.03	243.62		+			+							5	-503.4	1017.4	16.42	0.00	0.093
		241.76	209.05	274.47						+						4	-504.6	1017.6	16.62	0.00	0.070
		240.78	104.67	376.89			+									4	-504.8	1018.1	17.09	0.00	0.065
		232.16	209.84	254.47					+							4	-504.8	1018.1	17.10	0.00	0.065
		241.13	207.61	274.66					+	+						5	-504.6	1019.8	18.81	0.00	0.070
		253.61	242.67	264.54												2	-508.0	1020.1	19.09	0.00	0.000
**(F)**	**Edible crab**																				
		**39.92**	**-27.81**	**107.65**			**+**									**4**	**-196.3**	**401.7**	**0.00**	**0.80**	**0.223**
		58.77	-32.72	150.26	+		+					+				6	-196.1	406.4	4.73	0.08	0.231
		147.27	134.90	159.65					+							4	-199.5	408.1	6.41	0.03	0.104
		135.48	129.12	141.85												2	-202.0	408.3	6.63	0.03	0.000
		141.01	119.18	162.84					+	+						5	-199.3	410.1	8.44	0.01	0.114
		144.71	131.66	157.77	+											4	-200.6	410.2	8.53	0.01	0.061
		145.36	123.73	166.98						+						4	-200.7	410.4	8.76	0.01	0.056
		144.20	127.67	160.72		+										4	-200.7	410.4	8.78	0.01	0.055
		145.21	128.90	161.53		+			+							5	-199.5	410.4	8.79	0.01	0.107
		146.86	131.99	161.74	+				+					+		6	-199.2	412.6	10.99	0.00	0.116
		152.11	130.91	173.32	+	+					+					6	-199.9	414.1	12.45	0.00	0.087
		141.59	129.11	154.06				+								7	-198.8	414.6	12.99	0.00	0.132
		147.70	132.93	162.47				+	+							8	-197.6	415.1	13.49	0.00	0.179

	143.84	111.70	175.98	+					+					+	6	-200.5	415.3	13.61	0.00	0.063
	131.07	106.79	155.34	+			+					+			9	-196.5	416.2	14.54	0.00	0.216
	138.88	119.86	157.90		+		+								8	-198.7	417.5	15.80	0.00	0.135
	145.21	121.57	168.85				+		+						8	-198.7	417.5	15.82	0.00	0.135
**(G) European spiny lobster**																				
	**159.57**	**131.16**	**187.97**		**+**										**4**	**-127.3**	**264.2**	**0.00**	**0.40**	**0.173**
	125.37	119.59	131.16												2	-130.3	265.0	0.77	0.27	0.000
	157.41	127.54	187.29		+			+							5	-127.2	266.7	2.54	0.11	0.181
	101.75	14.03	189.48			+		+							4	-129.6	268.7	4.55	0.04	0.042
	162.10	132.89	191.31	+	+					+					6	-126.6	268.8	4.56	0.04	0.209
	133.80	116.44	151.15						+						4	-129.6	268.8	4.58	0.04	0.041
	127.52	113.30	141.74					+							4	-129.7	269.0	4.76	0.04	0.035
	129.62	118.67	140.58	+											4	-129.8	269.2	4.96	0.03	0.029
	131.77	109.45	154.09					+	+						5	-129.6	271.5	7.35	0.01	0.044
	133.63	115.57	151.69	+					+					+	6	-129.4	274.3	10.06	0.00	0.056
	108.27	13.95	202.59	+		+					+				6	-129.4	274.4	10.19	0.00	0.052
	127.57	112.69	142.44	+				+					+		6	-129.5	274.4	10.22	0.00	0.051
	147.75	106.40	189.11		+		+								9	-125.5	277.5	13.30	0.00	0.267
	119.74	82.76	156.73				+								8	-128.7	280.0	15.84	0.00	0.094
	104.43	44.29	164.58	+			+					+			9	-128.5	283.5	19.32	0.00	0.109
	119.38	81.47	157.29				+	+							9	-128.7	284.0	19.81	0.00	0.095
	120.76	76.74	164.78				+		+						9	-128.7	284.0	19.85	0.00	0.094
